# Cationic clustering influences the phase behaviour of ionic liquids

**DOI:** 10.1038/s41598-018-33176-6

**Published:** 2018-10-03

**Authors:** Thomas Niemann, Dimitri Zaitsau, Anne Strate, Alexander Villinger, Ralf Ludwig

**Affiliations:** 1 Universität Rostock, Institut für Chemie, Abteilung für Physikalische Chemie, Dr. -Lorenz-Weg 2, 18059 Rostock, Germany; 2 Universität Rostock, Institut für Chemie, Abteilung für Anorganische Chemie, Albert-Einstein-Str. 3a, 18059 Rostock, Germany; 3 Department Life, Light & Matter, University of Rostock, 18051 Rostock, Germany; 4 Leibniz-Institut für Katalyse an der Universität Rostock e.V., Albert-Einstein-Str. 29a, 18059 Rostock, Germany

## Abstract

“Unlike charges attract, but like charges repel”. This conventional wisdom has been recently challenged for ionic liquids. It could be shown that like-charged ions attract each other despite the powerful opposing electrostatic forces. In principle, cooperative hydrogen bonding between ions of like-charge can overcome the repulsive Coulomb interaction while pushing the limits of chemical bonding. The key challenge of this solvation phenomenon is to establish design principles for the efficient formation of clusters of like-charged ions in ionic liquids. This is realised here for a set of well-suited ionic liquids including the same hydrophobic anion but different cations all equipped with hydroxyethyl groups for possible H-bonding. The formation of H-bonded cationic clusters can be controlled by the delocalization of the positive charge on the cations. Strongly localized charge results in cation-anion interaction, delocalized charge leads to the formation of cationic clusters. For the first time we can show, that the cationic clusters influence the properties of ILs. ILs comprising these clusters can be supercooled and form glasses. Crystalline structures are obtained only, if the ILs are dominantly characterized by the attraction between opposite-charged ions resulting in conventional ion pairs. That may open a new path for controlling glass formation and crystallization. The glass temperatures and the phase transitions of the ILs are observed by differential scanning calorimetry (DSC) and infrared (IR) spectroscopy.

## Introduction

The pairing between opposite-charged ions is a well-accepted concept in chemistry and plays an important role for reactions in solution, macromolecular catalysis, biochemical hydrolysis and protein stability^1–5^. In contrast, establishing the theoretical foundation and experimental evidence for like-charge attraction in solution is a challenge. It seemingly contradicts conventional wisdom that like-charged ions can attract, despite the powerful opposing electrostatic forces: “Unlike charges attract, but like charges repel”. However, additional attractive forces between ions of like charge may attenuate the repulsive forces resulting in cationic or anionic cluster formation. Attractive interaction between ions of like charge has been observed for aqueous salt solutions of K/CsBr^6^, for guanidinium ions in water^7^, for tetraalkylammonium surfactants^8^, for oligopeptides and for DNA^9–11^. Recently, McNally *et al*. reported the self-assembly of molecular ions via like-charge ion interaction and through space defined organic domains^12^. Fatila *et al*. showed that hydrogen-bonded anions can stabilize each other inside macrocyclic hosts^13^. Both groups related the like-charge attraction to anti-electrostatic hydrogen bonds (AEHB) as suggested in Weinhold’s theoretical work^14,15^. So far, this phenomenon was only reported for large-scale structures, assemblies or stabilizing frameworks. Recently, we could show for ionic liquids that hydrogen bonding can overcome the repelling forces between ions of like-charge resulting in stable cationic clusters up to cyclic tetramers (ILs)^16–19^. Usually, the interaction between opposite-charged ions is typical for this unique liquid material, and determines its properties^20–28^. Moreover, we could show that the appearance of cationic clusters can be controlled by the interaction strength of the counterions. Strongly interacting anions suppress and weakly interacting anions promote the cationic cluster formation^29^. However, significant cationic cluster formation could be only observed at low temperatures, probably due to kinetic trapping.

In the present study, we demonstrate by the means of infrared (IR) spectroscopy that the presence of cationic clusters can be strongly supported or suppressed by specifically designed cations. The ability for cationic cluster formation can be referred to the shape, the charge distribution and the specific interaction site of the cations. By tuning the properties of the cations, we observe substantial cationic cluster formation already at room temperature. Here, the cationic clusters exist in equilibrium with the clusters of opposite-charged ions and ‘kinetic trapping’ is no longer a requirement for their existence^29^. For the first time we can also show that the presence of cationic clusters strongly influences the properties of ionic liquids. If substantial amount of cationic clusters is available, the ILs can be supercooled and form glasses at temperatures between 193 and 213 K. If cationic clusters are only present in trace amounts, the ILs can be crystallized by forming hydrogen-bonded ‘ion pairs’ between cations and anions solely^30^. The glass transition temperatures and the phase transition temperatures were measured by differential scanning calorimetry (DSC). If crystallization occurs, the infrared spectra show the typical sharp features of cation-anion interaction only and the broad redshifted bands of the cationic clusters completely disappear. For these ILs the structures could be obtained from X-ray crystallography^30^. The formation of clusters of like-charged ions is influenced by the shape and interaction strength of the ions and may provide a new path for controlling supercooling and crystallization.

## Results and Discussion

**Figure 1:**
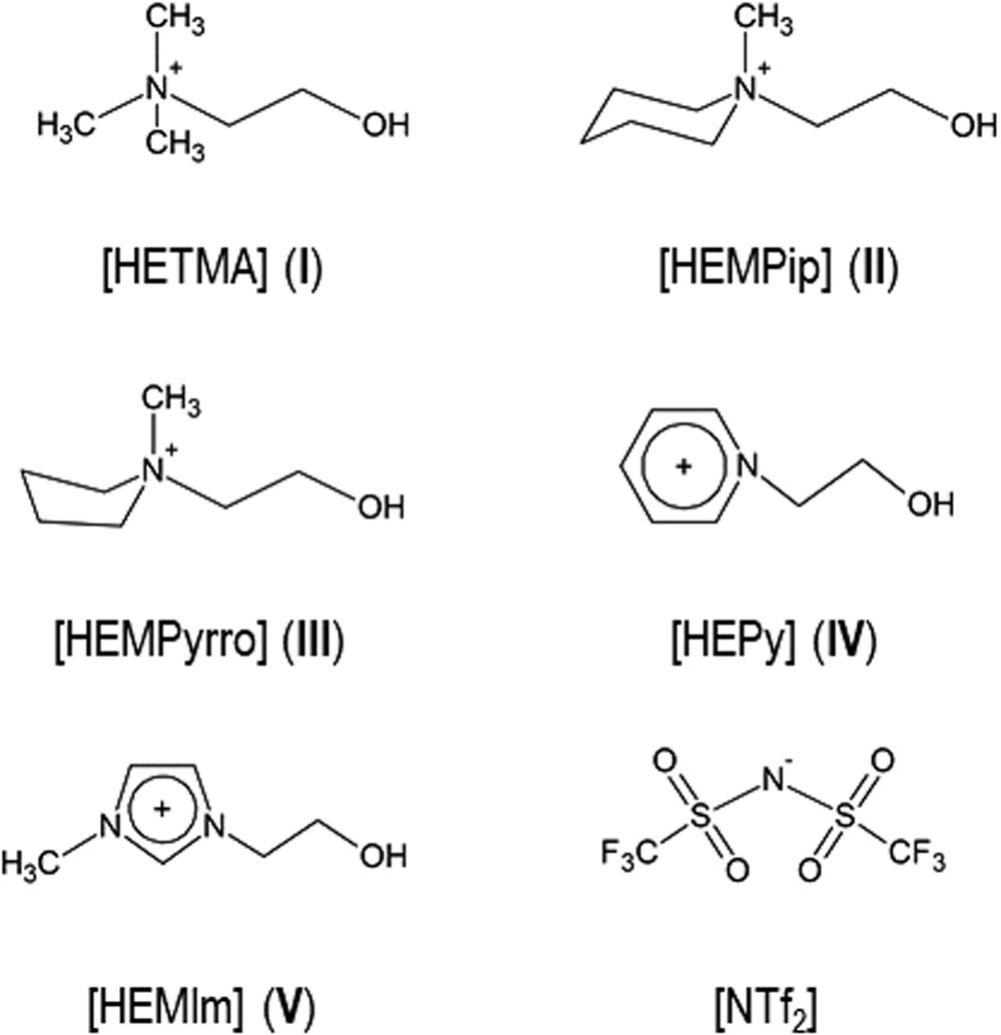
Structures of the cations [HETMA]^+^ (**I**), [HEMPip]^+^ (**II**), [HEMPyrro]^+^ (**III**), [HEPy]^+^ (**IV**), [HEMIm]^+^ (**V**) and the anion [NTf_2_]^−^ which is present in all ILs **I**–**V**.

First, we studied the formation and stability of cationic clusters for a set of specifically selected ionic liquids (see SI1). The ILs are based on ammonium, piperidinium, pyrrolidinium, pyridinium, and imidazolium cations all including a hydroxyethyl group for H-bond formation to the anion or to other cations resulting in cationic clusters. The cations are shown in Fig. 1. For all ILs, we have chosen the bis(trifluoromethylsulfonyl)imide [NTf_2_]^−^ as the counterion for two reasons. Firstly, this anion is flexible resulting in low viscosities and melting points, finally providing room temperature ionic liquids. Secondly, the relatively hydrophobic [NTf_2_]^−^ anion forms only weak hydrogen bonds to the cation and thus supports the formation of cationic clusters. The five ILs under investigations are [HETMA]NTf_2_ (**I**), [HEMPip]NTf_2_ (**II**), [HEMPyrro]NTf_2_ (**III**), [HEPy]NTf_2_ (**IV**), and [HEMIm]NTf_2_ (**V**) (see Fig. 1). The ILs **II**, **II** and **IV** were synthesized in our lab (see SI1), the ILs **I** and **V** were purchased form IoLiTec.

**Figure 2:**
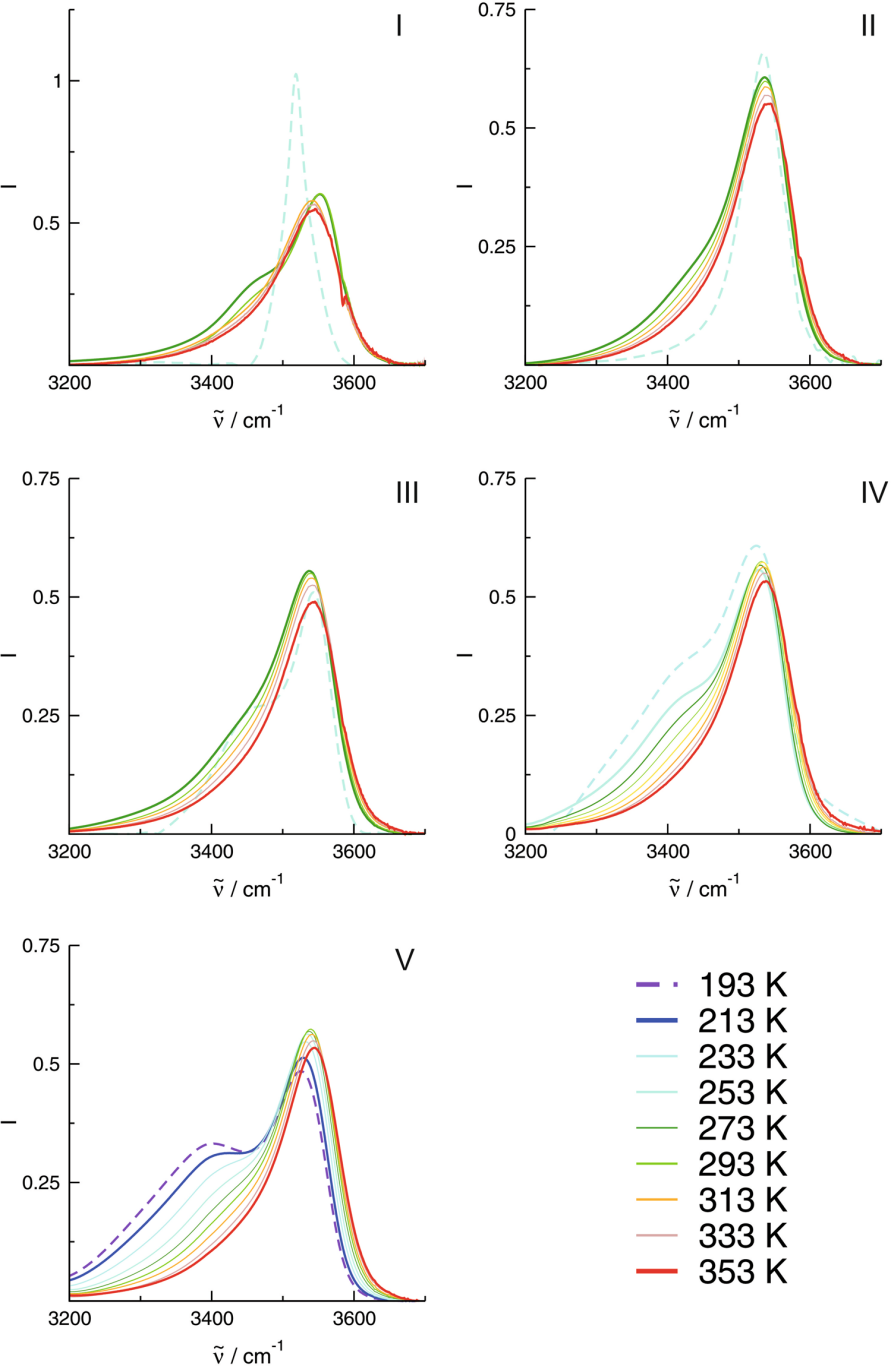
Infrared spectra in the O-H stretching region recorded for the ionic liquids (**a**) [HETMA]NTf_2_ (**I**), (**b**) [HEMPip]NTf_2_ (**II**), (**c**) [HEMPyrro]NTf_2_ (**III**), (**d**) [HEPy]NTf_2_ (**IV**) and (**e**) [HEMIm]NTf_2_ (**V**) as a function of temperature. The vibrational bands above 3500 cm^−1^ indicate hydrogen bonding between cation-anion, whereas the redshifted vibrational bands represent cationic clusters. In the sequence IL **I**–**V** the formation of cationic clusters strongly increases. The dashed lines indicate glass transition (**II**, **IV**, **V**) or phase transition (**I**, **II**) temperatures.

In respect to structure and charge distribution within the cation, the ILs can be divided into two groups. ILs **I**, **II** and **III** represent saturated compounds, characterized by positive charge localization at the nitrogen atom. In contrast, delocalized positive charge within the nitrogen heterocycle is typical for ILs **IV** and **V**. The transmission infrared spectra for all ILs **I**-**V** are shown as a function of temperature in Fig. 2. We started at the highest temperature of about 353 K for all compounds and went down below the phase transition temperature for each IL ranging between 253 K for ILs **I**, **II** and **III**, 213 K for IL **IV** and 193 K for IL **V**.

For all ILs **I**–**V** the observed OH stretch vibrational bands above 3500 cm^−1^ can be exclusively referred to the hydrogen bond enhanced OH^…^O=S cation-anion (**c**-**a**) interaction. In all compounds **I**–**V** these vibrational bands have almost similar frequencies and intensities at 353 K, which indicates that the **c-a** hydrogen bonds are independent of the nature of the cation (see SI2). For all ILs vibrational intensity is also observed below 3500 cm^−1^ but to different extent depending on the type of cation in the ILs **I**-**V**. These redshifted vibrational features can be assigned to the OH^…^OH hydrogen bonds among the cations (**c**-**c**) indicating the presence of cationic clusters (see Fig. 2). Recently, both structural motifs have been observed for isolated cationic complexes of IL **V** by using a combined approach of photodissociation mass spectrometry and double resonance techniques^31^.

For ILs **I** and **II** the IR spectra are mainly characterized by the vibrational bands at 3540 cm^−1^ indicating pure **c-a** interaction along the OH^…^O=S hydrogen bond. Only little redshifted contributions can be observed for these ILs. Below their melting points, the IR spectra in the OH frequency range become highly symmetric. (see SI3) Even the trace amounts of redshifted intensities disappear and the ionic interaction is described by **c-a** hydrogen bonds only.

**Figure 3:**
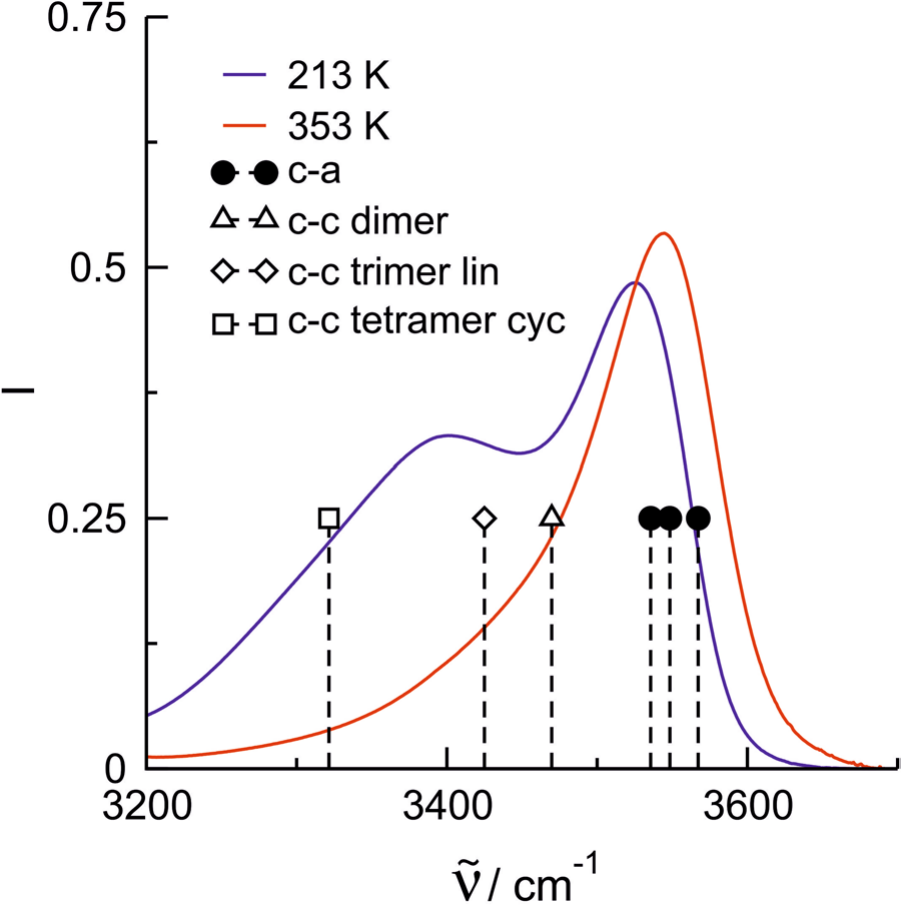
Infrared spectra in the O-H stretching region of the ionic liquid [HEMIm]NTf_2_] (**V**) at 213 (blue curve) and 353 K (red curve). The vibrational bands above 3500 cm^−1^ indicate hydrogen bonding between cation-anion (**c**-**a**), whereas the redshifted vibrational bands represent cationic (**c**-**c**) clusters. This interpretation is supported by frequency calculations at the B3LYP/6-31 + G* level of theory. We calculated dimers, trimers and tetramers of pure **c**-**a** and **c**-**c** clusters. The c-a frequencies lie above 3500 cm^−1^ throughout (filled symbols), whereas the c-c cluster frequencies are further redshifted with increasing c-c cluster size (open symbols). The comparison with the experimental spectra suggest that linear trimer and cyclic tetramers are need to represent the measure IR spectra. For each cluster averaged frequencies which are weighted with the calculated intensities.

The interpretation of the vibrational bands is supported by DFT calculated frequencies of **c-a** and **c-c** type clusters including one up to four ion pairs at the B3LYP-D3/6-31 + G* level of theory (see SI4)^32–35^. Figure 3 shows the spectrum of [HEMIm]NTf_2_] (**V**) at the lowest (213 K) and highest (353 K) temperatures. Additionally the calculated averaged frequencies of a dimer, trimer and tetramer of purely **c**-**a** bound clusters along with those of a dimer, linear trimer and cyclic tetramer of purely **c-c** bound clusters are shown. The calculated **c**-**a** frequencies lie clearly above 3500 cm^−1^, whereas the **c**-**c** cluster frequencies are further redshifted with increasing cluster size strongly supporting the existence of two types of cluster species. The agreement between the calculated and measured frequencies also underlines that the **c-c** hydrogen bonds are stronger than those in the **c-a** clusters. That is quite surprising because the **c-a** hydrogen bonds are additionally enhanced by Coulomb attraction, whereas the **c-c** hydrogen bonds have to compete with the repulsive Coulomb forces between like-charged ions. Overall, the comparison with the experimental spectra suggest that the existence of linear trimer and cyclic tetramers are required to represent the measured IR spectra.

**Figure 4:**
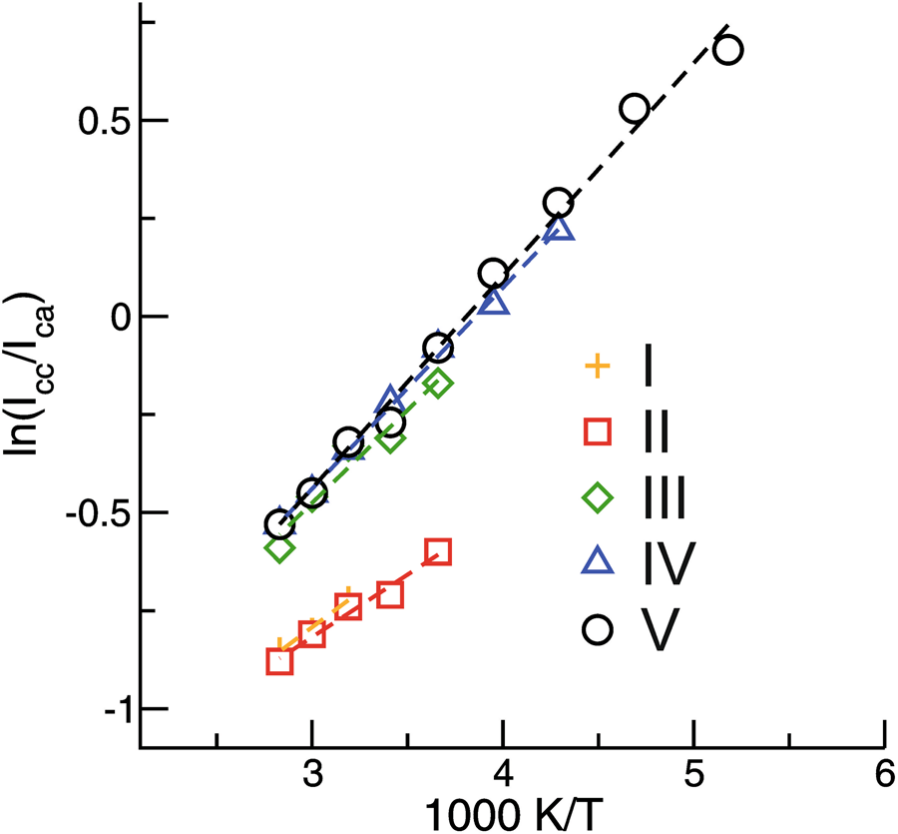
Plots of the natural logarithm of the **c-c** to **c-a** vibrational band intensity ratios versus inverse temperature taken from the measured spectra in Fig. 1 between 353 K and 193 K. *I*_cc_ and *I*_ca_ were obtained from the integral intensities left and right of the frequency position, where the deconvoluted vibrational bands for the **ca** and the **cc** species cross. The solid lines represent linear fits (R^2^ ≥ 0.98) with slopes indicating different enthalpies of cationic cluster formation.

We assume that the cation-anion (*I*_ca_) and the cation-cation (*I*_cc_) hydrogen bond intensities arise from cations of these two general classes of H-bonding configurations, for which relative populations are a function of absolute temperature only. Consequently, a plot of ln(*I*_cc_/*I*_ca_) versus 1/*T* should yield a straight line with a slope that is proportional to the average difference in energy between the two classes of H-bond configurations. All ILs show a linear dependence between ln(*I*_cc_/*I*_ca_) and 1/*T*. The slope is positive for all ILs indicating that the formation of the cationic clusters resembles an exothermic reaction (see Fig. 4). Obviously, we observe two types of cations involved in different types of clusters, **c-c** and **c-a**. The slopes for IL **I** and **II** are significantly smaller than those for the other ILs (1.6 vs 1.0). The low reaction enthalpy for the ILs comprising saturated cations with localized charge indicates that only small clusters are formed in accord with the small redshifted contributions in the IR spectra. The other ILs in particular **IV** and **V** form larger clusters with enhanced stability which cannot be destroyed in favor of crystallization.

**Figure 5:**
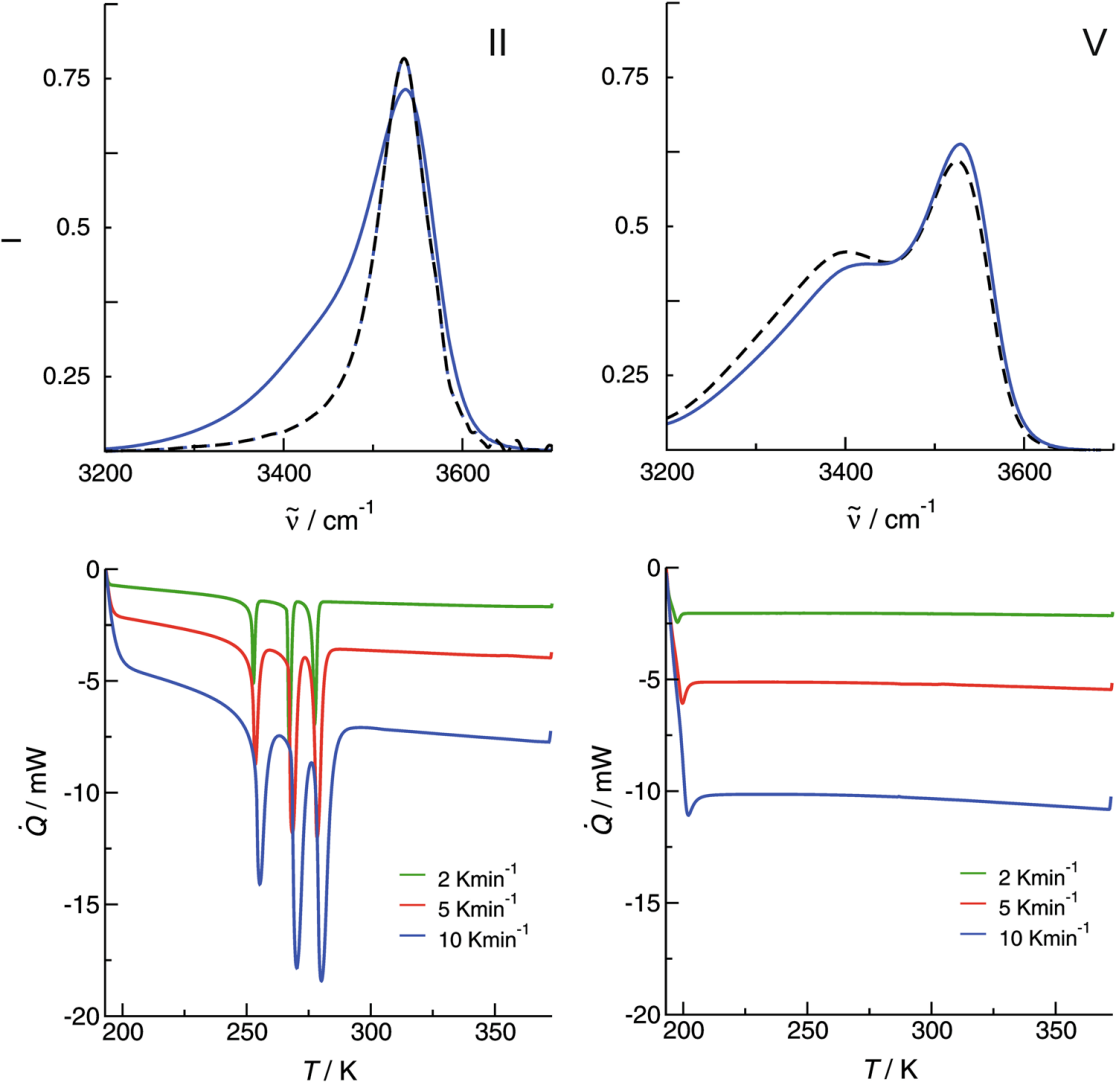
Infrared spectra in the O-H stretching region and DSC recorded for the ionic liquids (**a**) [HEMPip]NTf_2_ (**II**) and (**b**) [HEMIm]NTf_2_ (**V**) for the lowest (**a**) 253 K, (**b**) 193 K) and second lowest (**a**) 273 K, (**b**) 213 K) temperatures. The vibrational bands above 3500 cm^−1^ indicate hydrogen bonding between cation-anion, whereas the redshifted vibrational bands represent cationic clusters. For IL **II** the small amount of cationic clusters disappears upon freezing resulting in a symmetric vibrational band indicating pure **c**-**a** interaction. This behavior is in accord to the DSC traces, which show phase transitions in this temperature range. For IL **V** the amount of cationic clusters further increase with decreasing temperature. No change in the vibrational signature is observed in accord with the DSC traces.

The interpretation of the IR spectra at low temperatures is also supported by the DSC measurements (see also SI5). During cooling from 373 to 193 K at 2, 5 and 10 K·min^−1^, only a heat capacity change corresponding to the glass transition (*T*_g_) could be observed in the DSC profiles of IL **IV** and **V**. It means that the supercooled liquids are fairly stable. For ILs **I**, **II** and **III** complex phase transition with melting (*T*_fus_) and solid/solid phase transition (*T*_ss_) are observed. Figure 5 shows the DSC traces related to the temperature dependent IR spectra of ILs **II** and **V**. After phase transition, the IR spectra for IL **II** only exhibit **c-a** vibrational bands, whereas the minor **c-c** spectral features typical for the liquid phase completely disappear. Obviously, the disappearance of the cationic clusters and the presence of pure **c-a** bonded ion pairs are related to fusion at 276.2 K and two solid/solid phase transitions at 266.2 and 251.6 K, respectively. In contrast, the strong formation of cationic clusters in IL **V** is not changing the spectral features with decreasing temperature. The liquid can be supercooled and show glass transition between 195 and 198 K. From the combined IR and DSC experiments, we have clear evidence that the formation of cationic clusters prevents the ILs from crystallization and liquid/solid phase transition. The resulting material is a glass.

Both, IR and DSC experiments suggest that ILs **I** and **II** should give crystal structures. The crystal structure for IL **I** is known from the literature and shows one cation-anion pair in the asymmetric unit and four ion pairs in the unit cell^30^. As expected, the choline cation and the [NTf_2_]^−^ anion are held together by hydrogen bonds with an interatomic distance between the hydroxyl proton and the sulfonyl oxygen atom of about r(H…O=S) = 205 pm. Unfortunately, we failed to obtain the crystal structure for IL **II**. We found a low-temperature structure at 12 K (see SI6) that showed neither H-bonding between anions and cations, nor H-bonding among the cations. This structure showed a delocalized positive charge, which is spherically surrounded by the negative partial charges of the anions. The DCS traces show a second crystalline phase of IL **II** at 267 K between the low-temperature phase and the amorphous (glassy) phase (see Fig. 5). Probably due to high thermal motion and resulting decrease in scattering power, we were not able to solve the crystal structure of this phase.

Overall, the IR and DSC measurements clearly indicate different phase transition behavior for these two types of ILs. If substantial cationic clusters are formed, the liquid IL will supercool and becomes a glass at low temperatures as in ILs **IV** and **V**. No liquid/solid phase transition is observed. Usually, crystallization can be avoided only, if liquids are cooled sufficiently fast. Molecules will then arrange so slowly that they cannot adequately sample configurations in the available time allowed by the cooling rate. The liquid’s structure, therefore, appears ‘frozen’. The resulting material is a glass^36^. Here, we show that substantial cationic cluster formation prevents crystallization and glass formation for ILs. Hydrogen bonding immobilizes the cations and no solid configurations can be formed.

## Conclusions

In this work, we could show for a set of well-suited ionic liquids that the presence of like-charge attraction is governed by the charge delocalization of the cation. If the positive charge is delocalized and available to the counterion, cooperative hydrogen bonding between the like charges is possible resulting in cationic clusters up to cyclic tetramers. As shown in the IR spectra, these clusters are characterized by redshifted vibrational bands which increase in intensity with decreasing temperature. The ILs exhibiting substantial amounts of cationic clusters form glasses as shown by DSC measurements. The ILs showing only traces of these structures can be crystallized or solidified, resulting in pure cation-anion hydrogen bonds. The glass and liquid/solid phase transitions are also observed in the IR experiments. Obviously, the amount of cationic clusters influence the glass formation and crystallization. For the first time we could show, that like-charge attraction modifies the macroscopic properties of ILs. The formation of clusters of like-charged ions is influenced by the shape and interaction strength of the ions and may provide a new path for controlling crystallization and glass formation.

## Electronic supplementary material

Supplementary Information
