# Supplementary material for: Cationic clustering influences the phase behaviour of ionic liquids
Source: Sci Rep. 2018 Oct 3;8:14753. doi: 10.1038/s41598-018-33176-6 (PMC6170405; doi:10.1038/s41598-018-33176-6)
Supplement: Supplementary file 1 — Supplementary Information [file 41598_2018_33176_MOESM1_ESM.pdf]

Cationic clustering influences the phase behaviour of ionic liquids*

M. Sc. Thomas Niemann,[a] Dr. Dimitri Zaitsau,[a] Dr. Anne Strate,[a] Dr. Alexander Villinger,[b] and Prof. Dr. Ralf Ludwig[a,c,d]*

[a]Universität Rostock, Institut für Chemie, Abteilung für Physikalische Chemie
Dr.-Lorenz-Weg 1, 18059, Rostock (Germany)

[b]Universität Rostock, Institut für Chemie, Abteilung für Anorganische Chemie
Albert-Einstein-Str. 3a, 18059 Rostock (Germany)

[c]Department Life, Light & Matter, University of Rostock, 18051 Rostock (Germany)

[d]Leibniz-Institut für Katalyse an der Universität Rostock e.V.
Albert-Einstein-Str. 29a, 18059 Rostock (Germany)

*E-mail: ralf.ludwig@uni-rostock.de

Supplementary Information

Contents

SI1 **Synthesis of ILs II, III and IV**

SI2 **IR measurements and IR spectra at highest temperature 353 K**

SI3 **Melting points T_m or glass transition temperatures T_g of ILs I-V**

SI4 **DFT-D3 optimized geometries of [HEMim][NTf_2] and of [HEPy][NTf_2] clusters c-a and c-c, frequencies of [HEMim][NTf_2] clusters c-a and c-c**

SI5 **DSC study and thermal behavior**

SI6 **Single crystal preparation and X-ray Determination**

SI1 Synthesis of ILs **II**, **III** and **IV**

General synthesis

Synthesis of the onium salts: Equimolar amounts of the heterocyclic amine and 2-bromoethanol were mixed and heated up to 110 °C for 1h. Upon cooling, the mixture began to crystallize. The crude product was recrystallized from acetone/acetonitrile mixtures to obtain the colorless crystalline product.
Synthesis of the bis(trifluoromethanesulfonyl)imide: Equimolar amounts of the onium halide and lithium-bis(trifluoromethanesulfonyl)imide were mixed as aqueous solutions for 1h. Two phases were obtained, the lower one was washed several times with water until no residual bromine could be detected with silver nitrate solution. The obtained colorless liquids were dried for several hours in vacuum at 60 °C.

Synthesis of the specific compounds

Apart from the reactions in aqueous solutions all reactions were performed in a moisture-guarded assembly and reflux condenser was used while heating. The used solvents were dried with molecular sieves to a water content less than 50 ppm and distilled freshly. All starting materials used in the synthesis were purchased from SIGMA ALDRICH and dried by conventional methods for the use in moisture-free reactions. The ionic liquids and 1-(2-hydroxyethyl)-1,1,1-trimethylammonium-bis(trifluoromethanesulfonyl)imide (**I**) and 1-(2-hydroxyethyl)-3-methylimidazolium-bis(trifluoromethanesulfonyl)imide (**V**) were purchased from IoLiTec.

1-(2-Hydroxyethyl)-1-methylpiperidinium-bromide [HEMPip]Br

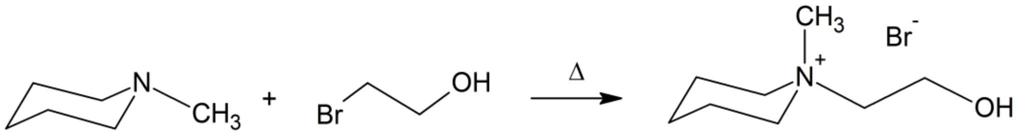

32.661 g of 2-bromoethanol (261 mmol, 19 ml) were added to a solution of 16.378 g of N-methylpiperidine (165 mmol, 20 ml) and 40 ml ethanol at room temperature. The mixture was refluxed for 2 h. After cooling to room temperature the ethanol and the residual 2-bromoethanol were removed in vacuum. The liquid crude product begins to crystallize by addition of 40 ml of acetone. The white precipitate was washed three times with dry acetone. The product was dried for 4 h in vacuum at 60 °C. (**[HEMPip]Br**) was obtained in 89 % yield.
EA % cal. (exp.): C 42.87 (42.65); H 8.10 (8.06); N 6.25 (6.11). ^{1}**H-NMR**(298.2 K, DMSO-d$_6$, 300.13 MHz, [ppm]): δ = 1.48-1.58 (m, 2H, C(4)H_2); 1.74-1.84 (m, 4H, C(2; 6)H_2); 3.08 (s, 3H,

CH_3); 3.30-3.47 (m, 6H, C(3; 5)H_2 +CH$_2$−CH_2−OH); 3.81-3.88 (m, 2H, CH_2−CH$_2$−OH), 5.26 (t, 1H, O*H*). ^{13}**C-NMR** (298 K, DMSO-d$_6$, 75.46 MHz, [ppm]): δ = 20.48 (s, *C*(4)); 19.24 (s, *C*(3; 5)); 48.07 – 48.27 (m, *C*H$_3$); 54.49 (s, *C* H$_2$−CH$_2$−OH); 60.77 – 60.92 (m, CH$_2$−*C*H$_2$−OH); 63.72 – 63.93 (m, *C*(2; 6)).

1-(2-Hydroxyethyl)-1-methylpiperidinium-bis(trifluoromethanesulfonyl)imide [HEMPip]NTf$_2$ (II)

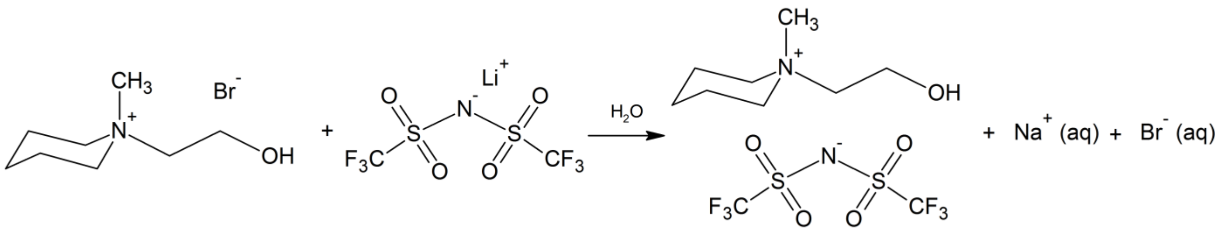

9.030 g (**II**) (40 mmol) were solved in 10 ml H_2O and added to a solution of 11.518 g LiNTf$_2$ (40 mmol) in 10 ml H_2O. The mixture was stirred for 1h. During this time two phases were formed. The lower phase was washed several times with water until no residual bromine could be detected with silver nitrate solution. The thus obtained colorless liquid was dried for 6 h at 110 °C in vacuum. Yield: 68 %.

^{1}**H-NMR**(300 K, DMSO-d$_6$, 250.13 MHz, [ppm]): δ = 1.46-1.57 (m, 2H, C(4)H_2); 1.71-1.82 (m, 4H, C(2; 6)H_2); 3.05 (s, 3H, CH_3); 3.26-3.44 (m, 6H, C(3; 5)H_2 CH$_2$−CH_2−OH); 3.78-3.86 (m, 2H, CH_2−CH$_2$−OH), 5.24 (t, 1H, O*H*). ^{13}**C-NMR**(298 K, DMSO-d$_6$, 75.6 MHz, [ppm]): δ = 20.48 (s, *C*(4)); 19.24 (s, *C*(3; 5)); 48.07 – 48.27 (m, *C*H$_3$); 54.49 (s, *C*H$_2$−CH$_2$−OH); 60.77 – 60.92 (m, CH$_2$−*C*H$_2$−OH); 63.72 – 63.93 (m, *C*(2; 6)); 119.21 (q, *C*F$_3$). ^{19}**F-NMR**(300 K, DMSO-d$_6$, 235.36 MHz, [ppm]): δ = −78.79 (s, C*F*$_3$). **IR** (Transm., CaF$_2$-Window, 12 μm-Spacer, 20 °C, 128 Scans, [cm^{-1}]): 3536 (w); 2958 (w); 2882 (w); 1846 (vw); 1797 (vw); 1471 (w); 1349 (s); 1332 (m); 1195 (s); 1138 (s); 1086 (w); 1056 (s); 986 (w).

1-(2-Hydroxyethyl)-1-methylpyrrolidinium-bromide [HEMPyrro]Br

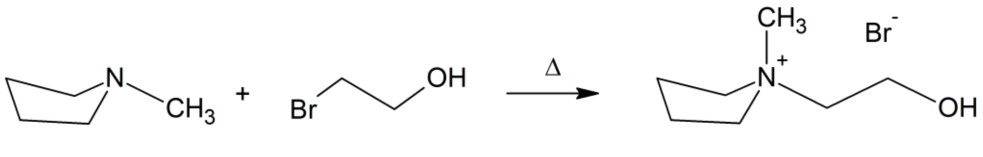

15.732 g N-Methylpyrrolidine (185 mmol; 20 ml) were mixed with 35.732 g 2-bromoethanol (286 mmol; 20 ml) at room temperature. The mixture was heated slowly up to 100 °C. When the reaction begins, the mixture turns brown. After 1 h at this temperature, the mixture was allowed

to cool down. Excess 2-bromoethanol and unreacted N-Methylpyrrolidine are then removed in vacuum. The brownish cristalline crude product was recristallized from 1400 ml of an acetone/acetonitrile mixture 3:1. The colorless product **([HEMPyrro]Br)** is obtained in the form of needle-shaped crystals. Yield 66,4%.

EA % cal. (exp.): C 40.01 (39.64); H 7.68 (7.75); N 6.67 (6.46). ^{1}**H-NMR**(298.2 K, DMSO-d$_6$, 300.13 MHz, [ppm]): δ = 2.03-2.13 (m, 4H, C(3; 4)H_2); 3.6 (s, 3H, CH_3); 3.43-3.48 (m, 2H, CH$_2$−CH_2−OH); 3.49-3.56 (m, 4H, C(2; 5)H_2); 3.79-3.87 (m, 2H, CH_2−CH$_2$−OH), 5.27 (t, 1H, O*H*). ^{13}**C-NMR** (298 K, DMSO-d$_6$, 75.46 MHz, [ppm]): δ = 20.81 (s, *C*(3; 4)); 47.83 – 48.02 (m, *C*H$_3$); 55.46 (s, *C*H$_2$−CH$_2$−OH); 64.09 – 64.26 (m, CH$_2$−*C*H$_2$−OH); 64.47 -64.61 (m, *C*(2; 5)). **IR** (ATR, 30 °C, 128 Scans, [cm^{-1}]): 3326 (vs); 3280 (vs); 3013 (m); 2963 (w); 2941 (w); 2887 (w); 1460 (m); 1395 (w); 1371 (w); 1305 (w); 1238 (w); 1179 (vw); 1081 (m); 1037 (w); 996 (m); 954 (w); 936 (m); 900 (w); 873 (w); 814 (w).

1-(2-Hydroxyethyl)-1-methylpyrrolidinium-bis(trifluoromethanesulfonyl)imide [HEMPyrro]NTf$_2$ (III)

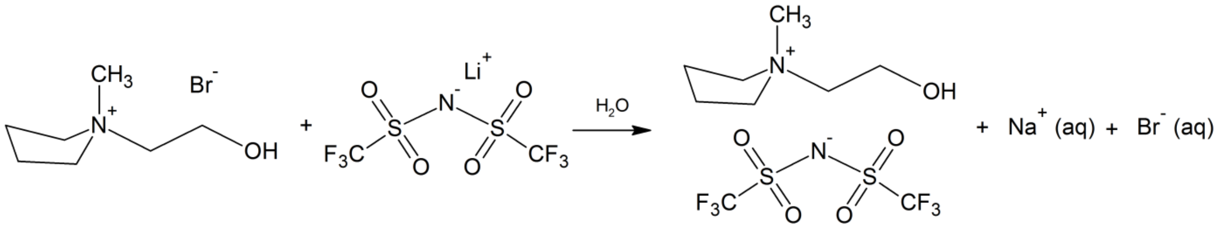

4.201 g (**7**) (20 mmol) dissolved in 10 ml H$_2$O were mixed with 5.739 g LiNTf$_2$ (20 mmol) dissolved in 10 ml H$_2$O. The mixture was stirred for 1 h. During this time two phases were formed. The lower one was washed several times with water until no residual bromine could be detected with silver nitrate solution. The thus obtained colorless liquid was dried 6 h at 110°C in vacuum. Yield: 63 %.

^{1}**H-NMR**(298.2 K, DMSO-d$_6$, 300.13 MHz, [ppm]): δ = 2.02-2.13 (m, 4H, C(3; 4)H_2); 3.06 (s, 3H, CH_3); 3.39-3.45 (m, 2H, CH$_2$−CH_2−OH); 3.49-3.56 (m, 4H, C(2; 5)H_2); 3.79-3.87 (m, 2H, CH_2−CH$_2$−OH), 5.26 (t, 1H, O*H*). ^{13}**C-NMR** (298 K, DMSO-d$_6$, 75.46 MHz, [ppm]): δ = 20.81 (s, *C*(3; 4)); 47.83 – 48.02 (m, *C*H$_3$); 55.46 (s, *C*H$_2$−CH$_2$−OH); 64.09 – 64.26 (m, CH$_2$−*C*H$_2$−OH); 64.47 – 64.61 (m, *C*(2; 5)); 119.43 (q, *C*F$_3$). ^{19}**F-NMR** (298.2 K, DMSO-d$_6$, 282.40 MHz, [ppm]): δ = −78.73 (s, C*F*$_3$). **IR** (Transm., CaF$_2$-Window, 12 μm-Spacer, 20 °C, 128 Scans, [cm^{-1}]): 3539 (w); 3044 (vw); 2980 (w); 2903 (w); 1849 (vw); 1799 (vw); 1701 (vw); 1470 (w); 1431 (w); 1355 (vs); 1355 (m); 1199 (vs); 1138 (s); 1054 (s); 998 (w); 986 (w).

1-(2-Hydroxyethyl)pyridinium-bromide [HEPy]Br

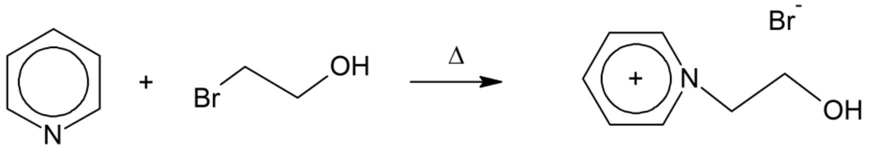

At room temperature equimolar amounts of pyridine (8.977 g; 113 mmol; 9.6 ml) and 2-bromoethanol (14.929 g; 113 mmol; 14.9 ml) were mixed and heated slowly up to 110 °C. When the reaction begins, the solution turns brown and starts to crystallize. The mixture was cooled to room temperature. The crude product was recrystallized from about 330 ml acetonitrile. The product (**[HEPy]Br**) was obtained as rod-shaped colorless crystals. Yield 89 %.

EA % cal. (exp.): C 41.20 (41.22); H 4.94 (4.92); N 6.86 (6.76). ^{1}**H-NMR**(298.2 K, DMSO-d$_6$, 300.13 MHz, [ppm]): δ = 3.86 (dd, 2H, CH$_2$−C*H*$_2$−OH); 4.67-4.74 (m, 2H, C*H*$_2$−CH$_2$−OH); 5.25(t, 1H, O*H*); 8.14-8.21 (m, 2H, *m*-C*H*); 8.60-8.67 (m, 1H, *p*-C*H*); 9.03-9.09 (m, 2H, *o*-C*H*). ^{13}**C-NMR**(298 K, DMSO-d$_6$, 75.46 MHz, [ppm]): δ = 59.98 (s, CH$_2$−*C*H$_2$−OH); 63.04 (s, *C*H$_2$−CH$_2$−OH); 127.67 (s, *m*-*C*H); 145.15 (s, *p*-*C*H); 145.53 (s, *o*-*C*H).

1-(2-Hydroxyethyl)pyridinium-bis(trifluoromethanesulfonyl)imide [HEPy]NTf$_2$ (**IV**)

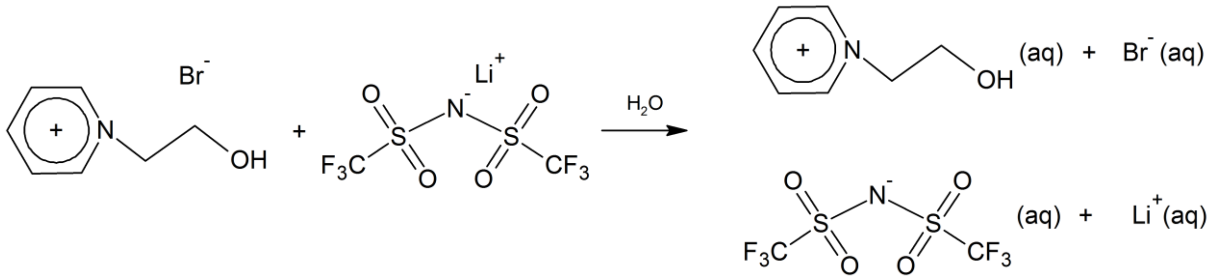

3.220 g (**[HEPy]Br**) (16 mmol) solved in 2 ml H$_2$O were added to a solution of 4.576 g lithium-bis(trifluoromethylsulfonyl)imide LiNTf$_2$ (16 mmol) in 2.5 ml H$_2$O. The mixture was stirred for 1 h. Two phases were obtained, the lower one was washed several times with water until no residual bromine could be detected with silver nitrate solution. The obtained colorless liquid of (**[HEPy]NTf**$_2$) was dried for 8 h in vacuum at 60 °C. Yield 57 %.

EA % cal. (exp.): C 26.73 (26.14); H 2.49 (2.41); N 6.93 (6.54); S 15.86 (15.50). ^{1}**HNMR**(298.2 K, DMSO-d$_6$, 300.13 MHz, [ppm]): δ = 3.88 (dd, 2H, CH$_2$−C*H*$_2$−OH); 4.65- 4.71 (m, 2H, C*H*$_2$−CH$_2$−OH); 5.22(t, 1H, O*H*); 8.12-8.20 (m, 2H, *m*-C*H*); 8.57-8.64 (m, 1H, *p*-C*H*); 9.00-9.05 (m, 2H, *o*-C*H*). ^{13}**C-NMR**(298 K, DMSO-d$_6$, 75.46 MHz, [ppm]): δ = 59.95 (s, CH$_2$−*C* H$_2$−OH); 63.01 (s, *C*H$_2$−CH$_2$−OH); 119.43 (q, *C*F$_3$); 127.65 (s, *m*-*C*H); 145.12 (s, *p*-*C*H);

145.50 (s, *o*-*C*H). ^{19}**F-NMR**(298 K, DMSO-d_6, 282.40 MHz, [ppm]): $\delta = -78.81$ (s, C*F*$_3$). **IR** (Transm., CaF_2-Window, 12 μm Spacer, 20 °C, 128 Scans, [cm^{-1}]): 3528 (vw); 3141 (vw); 3097 (vw); 3074 (vw); 2972 (vw); 2951 (vw); 2893 (vw); 2857 (vw); 1936 (vw); 1850 (vw); 1741 (vw); 1638 (w); 1585 (vw); 1502 (vw); 1491 (m); 1451 (vw); 1352 (vs); 1200 (s); 1136 (s); 1060 (s).

SI2 IR measurements and IR spectra at highest temperature 353 K

IR measurements

Mid infrared (MIR) measurements were performed with a Bruker Vector 22 FTIR spectrometer. An L.O.T.-Oriel variable-temperature cell equipped with CaF_2 windows having a path length of 12 μm was used for the variable-temperature experiments between 213 and 353 K. Cooling of the cell is achieved by means of a cooling dewar with liquid ethanol/nitrogen mixture. For each spectrum 128 scans were recorded at a spectral resolution of 1 cm^{-1}.

IR spectra at highest temperature 353 K

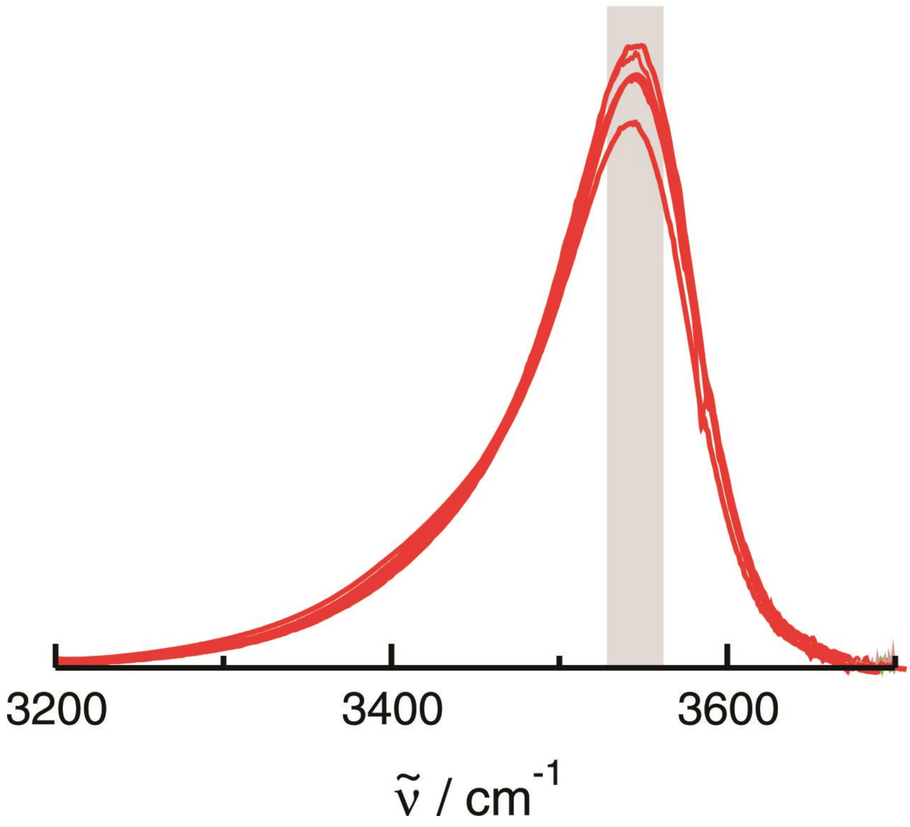

SI Fig. 1 Infrared spectra of the ILs **I-V** for the highest temperature at 353 K. It is observed that all ILs exclusively show **c-a** vibrational bands with almost similar frequencies and intensities.

SI3 Melting points T_m or glass transition temperatures T_g of ILs I-V

Cation	Anion	$T_m(CH_3)$	$T_m(OH)$	ΔT_m
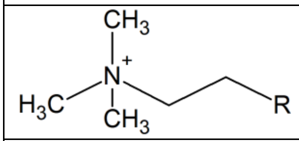	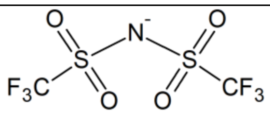	290-292 [1,2]	300,5	8,5-10,5
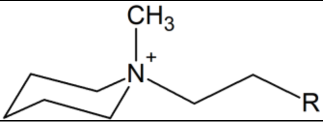	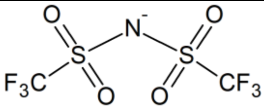	281 – 284 [2]	266,7	-14,3-(-)17,3
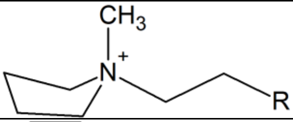	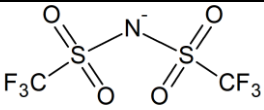	282-285 [3,4]	250,2	-35,0
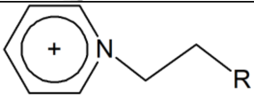	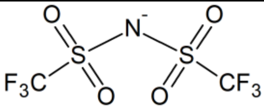	317,6	200,4 (glastr.)	(-115,1)
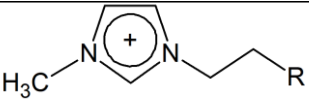	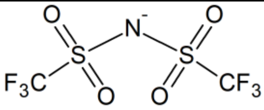	<195 (glastr.) [4]	< 195 (glastr.)	-

Table SI1 Melting points Tm and glass transition temperatures Tg of ILs I-V in comparison to literature data for the related alkylated ILs [1-4]. Therein the OH functional groups are replaced by methyl groups preventing the formation of hydrogen bonds. It is notable that the ILs with substantial cationic cluster formation (hydrogen bonding) have lower melting points.

[1] M. Yoshizawa-Fujita, Y. Kousa, K. Kidena, A. Ohira, Y. Takeoka, M. Rikukawa, *Phys. Chem. Chem. Phys.*, **2011**, 13, 13427–13432

[2] S. Katsuta, Y. Shiozawa, K. Imai, Y. Kudo, Y. Takeda, *J. Chem. Eng. Data*, **2010**, 55, 1588–1593

[3] G. B. Appetecchi, M. Montanino, D. Zane, M. Carewska, F. Alessandrini, S. Passerini, *Electrochimica Acta*, **2009**, 54, 1325–1332

[4] U. L. Bernard, E. I. Izgorodina, D. R. MacFarlane, *J. Phys. Chem. C*, **2010**, 114, 20472–20478

SI4 DFT-D3 optimized geometries of [HEMim][NTf_2] and [HEPy][NTf_2] clusters c-a and c-c, frequencies of [HEMim][NTf_2] clusters c-a and c-c

The IL clusters have been calculated at the DFT level B3LYP, using the internal stored 6-31+G* basis set of the Gaussian 09 program.[1] Grimme's DFT-D3 method was applied for calculating dispersion forces.[1-4]

Literature

[1] Gaussian 09, Revision B.01, M. J. Frisch, G. W. Trucks, H. B. Schlegel, G. E. Scuseria, M. A. Robb, J. R. Cheeseman, G. Scalmani, V. Barone, G. A. Petersson, H. Nakatsuji, X. Li, M. Caricato, A. Marenich, J. Bloino, B. G. Janesko, R. Gomperts, B. Mennucci, H. P. Hratchian, J. V. Ortiz, A. F. Izmaylov, J. L. Sonnenberg, D. Williams-Young, F. Ding, F. Lipparini, F. Egidi, J. Goings, B. Peng, A. Petrone, T. Henderson, D. Ranasinghe, V. G. Zakrzewski, J. Gao, N. Rega, G. Zheng, W. Liang, M. Hada, M. Ehara, K. Toyota, R. Fukuda, J. Hasegawa, M. Ishida, T. Nakajima, Y. Honda, O. Kitao, H. Nakai, T. Vreven, K. Throssell, J. A. Montgomery, Jr., J. E. Peralta, F. Ogliaro, M. Bearpark, J. J. Heyd, E. Brothers, K. N. Kudin, V. N. Staroverov, T. Keith, R. Kobayashi, J. Normand, K. Raghavachari, A. Rendell, J. C. Burant, S. S. Iyengar, J. Tomasi, M. Cossi, J. M. Millam, M. Klene, C. Adamo, R. Cammi, J. W. Ochterski, R. L. Martin, K. Morokuma, O. Farkas, J. B. Foresman, and D. J. Fox, Gaussian, Inc., Wallingford CT, 2016.

[2] S. Grimme, J. Antony, S. Ehrlich, H. Krieg, *J. Chem. Phys.* **2010**, 132, 154104.

[3] S. Ehrlich, J. Moellmann, W. Reckien, T. Bredow, S. Grimme**,** *ChemPhysChem.* **2011**, 12, 3414-3420.

[4] S. Grimme, A. Jansen, *Chem Rev.* **2016**, 116, 5105-5154.

[HEMim][NTf_2]

monomer c-a

```
*** imOH_NTf2_b3lyp_6-31+Gp_monomer_D3.g09, E(RB3LYP) =  -2247.22019498

0 1
      6           0       -0.859389    2.884991   -0.829451
      9           0       -1.756866    2.642463   -1.790148
     16           0       -0.267410    1.274074   -0.054327
      7           0       -1.695293    0.796611    0.515103
     16           0       -1.874426   -0.628140    1.244957
      6           0       -2.561453   -1.724561   -0.131269
      9           0       -1.618955   -1.912632   -1.086405
      9           0        0.210897    3.486766   -1.374895
      9           0       -1.393004    3.692942    0.087540
      8           0        0.694878    1.674209    0.999298
      8           0        0.285907    0.476272   -1.168254
      8           0       -0.599189   -1.299862    1.595419
      8           0       -2.949374   -0.563600    2.226544
      9           0       -3.638312   -1.180584   -0.701854
      9           0       -2.888089   -2.926595    0.367812
      8           0        3.491725    1.499841    1.165745
      6           0        3.592370    0.463354    2.116177
      6           0        2.683098   -0.737519    1.821157
      7           0        2.749595   -1.147027    0.401680
      6           0        1.733128   -1.721694   -0.250593
      1           0        2.968939   -1.588849    2.448946
      1           0        1.638210   -0.490859    2.016114
      1           0        3.345932    0.801469    3.134552
      1           0        4.645398    0.157188    2.124732
      1           0        2.549874    1.749179    1.060994
      6           0        3.782733   -0.929583   -0.494402
      7           0        2.084107   -1.896517   -1.527642
      6           0        3.363891   -1.397151   -1.704566
      1           0        0.768232   -1.938179    0.185999
      6           0        1.164353   -2.300821   -2.592515
      1           0        1.734485   -2.783836   -3.388192
      1           0        0.433702   -3.001590   -2.187878
      1           0        0.647098   -1.414105   -2.966873
      1           0        3.849675   -1.403354   -2.667728
      1           0        4.693957   -0.431713   -0.210417
```

dimer c-a

```
*** imOH_NTf2_b3lyp_6-31+Gp_dimer_D3.g09, E(RB3LYP) =  -4494.48143508

0 1
      6           0       -4.669937   -2.425265   -0.303709
      9           0       -5.792641   -1.817118   -0.688244
     16           0       -3.430458   -1.193303    0.400492
      7           0       -3.293352   -0.310198   -0.945084
     16           0       -2.378425    1.013649   -1.005209
      6           0       -3.580134    2.424773   -0.640622
      9           0       -4.154356    2.264415    0.560614
      9           0       -4.955612   -3.317947    0.653109
      9           0       -4.139141   -3.077928   -1.354660
      8           0       -2.230385   -2.021922    0.698549
      8           0       -4.080312   -0.551697    1.549186
```

8	0	-1.367052	1.118344	0.056417
8	0	-1.984587	1.248686	-2.398253
9	0	-4.533626	2.497627	-1.573347
9	0	-2.894391	3.588029	-0.627630
8	0	-1.580110	-1.978389	3.533673
6	0	-0.215910	-1.591891	3.498354
6	0	0.078850	-0.476587	2.499902
7	0	-0.591658	0.800389	2.807571
6	0	-0.078564	1.989083	2.472761
8	0	1.716194	0.577543	0.060338
16	0	2.769497	1.608469	0.063403
7	0	4.290560	1.096037	0.040313
16	0	4.716304	-0.425578	-0.317110
6	0	4.694964	-1.241520	1.386900
9	0	3.475711	-1.103776	1.954894
6	0	2.587964	2.455490	-1.610935
9	0	1.330636	2.899121	-1.758784
8	0	2.631502	2.707478	1.032953
9	0	3.433014	3.478439	-1.737184
9	0	2.838609	1.563190	-2.598532
8	0	6.113637	-0.447545	-0.729467
8	0	3.723232	-1.216641	-1.072909
9	0	4.935225	-2.565245	1.254067
9	0	5.608638	-0.718147	2.200327
8	0	2.428585	-4.319040	0.450344
6	0	1.813272	-3.803308	-0.718613
6	0	0.941130	-2.592127	-0.355785
7	0	0.324430	-1.998917	-1.549309
6	0	0.897446	-1.068352	-2.323130
1	0	1.150747	-0.277114	2.460567
1	0	-0.246158	-0.754424	1.496124
1	0	0.437303	-2.438649	3.234867
1	0	0.040866	-1.277606	4.515557
1	0	-1.867386	-2.211655	2.629290
1	0	0.133137	-2.881264	0.318176
1	0	1.547478	-1.817966	0.113302
1	0	1.193430	-4.606469	-1.131835
1	0	2.553942	-3.508081	-1.472468
1	0	3.225742	-3.799420	0.639886
7	0	0.070623	-0.796158	-3.339244
1	0	1.863284	-0.627791	-2.135792
6	0	0.277381	0.241005	-4.351547
6	0	-0.904643	-2.334583	-2.090446
6	0	-1.068361	-1.572699	-3.207714
1	0	-1.567691	-3.027293	-1.600144
1	0	-1.907387	-1.470141	-3.876462
1	0	0.075528	-0.179053	-5.339459
1	0	-0.398281	1.072314	-4.142577
1	0	1.311980	0.579797	-4.301790
6	0	-1.910343	0.977509	3.197251
7	0	-1.015097	2.925619	2.654103
6	0	-2.172263	2.312022	3.106661
1	0	0.910946	2.168837	2.076561
6	0	-0.864468	4.336842	2.301944
1	0	-1.132515	4.961332	3.157624
1	0	0.175163	4.519516	2.024935
1	0	-1.511726	4.564859	1.451125
1	0	-3.076122	2.867806	3.299659
1	0	-2.534070	0.140451	3.463235

trimer c-a

*** imOH_NTf2_b3lyp_6-31+Gp_trimer_D3.g09, E(RB3LYP) = -6741.74047200

0 1

7	0	4.396355	0.056833	3.330400
6	0	4.575263	0.492552	2.077754
7	0	5.451349	-0.317863	1.469351
6	0	5.828783	-1.316502	2.351989
6	0	5.175545	-1.072601	3.523312
6	0	5.799641	-0.206167	0.039377
6	0	7.162191	-0.787775	-0.318861
8	0	7.236482	-2.196268	-0.142921
6	0	3.498011	0.657912	4.317523
8	0	2.583418	-0.640850	0.211688
16	0	1.633948	-1.765753	0.146793
6	0	1.314862	-2.219436	1.962432
9	0	0.730951	-3.423317	2.042289
7	0	2.190402	-3.090250	-0.560745
16	0	3.747545	-3.529711	-0.445226
6	0	3.512142	-5.295549	-1.057621
9	0	4.718245	-5.876646	-1.104002
8	0	4.618417	-2.895283	-1.463999
8	0	4.283123	-3.616624	0.919658
9	0	2.976246	-5.303182	-2.285461
9	0	2.724514	-5.983343	-0.226582
8	0	0.279212	-1.523014	-0.371276
9	0	0.490247	-1.306453	2.504760
9	0	2.450485	-2.235531	2.671447
6	0	3.580591	-0.008346	-2.789320
6	0	4.146664	0.997412	-3.801679
8	0	5.454077	1.370478	-3.399994
7	0	2.178752	-0.362706	-3.043270
6	0	1.129592	0.383557	-2.669462
7	0	0.009864	-0.250569	-3.021631
6	0	0.347475	-1.444302	-3.633108
6	0	1.709825	-1.515324	-3.649050
6	0	-1.345392	0.171879	-2.651456
8	0	0.463999	1.922921	-0.139719
16	0	1.238303	2.440683	0.994696
7	0	2.782041	2.838229	0.685935
16	0	3.358784	3.274715	-0.758964
8	0	2.441197	3.184208	-1.903225
8	0	1.246470	1.695457	2.259979
6	0	0.364176	4.056615	1.439027
9	0	1.095709	4.762673	2.311481
9	0	0.148273	4.792045	0.345220
9	0	-0.817094	3.755559	2.002887
8	0	4.702881	2.676818	-0.905195
6	0	3.736447	5.110244	-0.536034
9	0	4.315396	5.567754	-1.652406
9	0	2.609848	5.793704	-0.310733
9	0	4.570117	5.284313	0.496495
9	0	-4.098351	2.988124	0.251011
6	0	-5.228022	2.640385	-0.406422
9	0	-6.264193	2.782821	0.431844
9	0	-5.386957	3.465837	-1.444631
16	0	-5.071618	0.846421	-0.968824
8	0	-3.970382	0.877358	-1.939070
8	0	-4.859205	0.114013	0.293742
7	0	-6.447760	0.561027	-1.745667
16	0	-7.761376	-0.063223	-1.028338
6	0	-7.513705	-1.918197	-1.300240
9	0	-7.497208	-2.220952	-2.602125

8	0	-8.938879	0.268894	-1.821586
8	0	-7.822611	0.067864	0.440575
9	0	-6.346004	-2.324247	-0.753018
9	0	-8.508416	-2.600389	-0.706754
8	0	-6.483038	-0.251192	2.889223
6	0	-6.616342	-1.651364	2.745773
6	0	-5.384033	-2.316328	3.358485
7	0	-4.161467	-1.953439	2.627457
6	0	-3.469076	-2.751562	1.732711
6	0	-2.475972	-1.985244	1.196493
7	0	-2.577615	-0.733750	1.771796
6	0	-3.610749	-0.732173	2.617757
6	0	-1.797975	0.450599	1.393132
1	0	-2.464227	1.174810	0.922796
1	0	5.755509	0.852368	-0.217261
1	0	5.021082	-0.726720	-0.516104
1	0	7.366086	-0.499268	-1.360340
1	0	7.949189	-0.358222	0.310108
1	0	6.537055	-2.613494	-0.680225
1	0	4.158234	-0.933383	-2.786199
1	0	3.620230	0.413858	-1.786560
1	0	4.232471	0.540974	-4.793239
1	0	3.480187	1.867430	-3.873251
1	0	5.367676	1.971669	-2.636303
1	0	1.185598	1.316873	-2.128912
1	0	2.381421	-2.284904	-3.995776
1	0	-0.403636	-2.139669	-3.973431
1	0	-1.985523	0.199088	-3.533421
1	0	-1.749355	-0.529594	-1.922033
1	0	-1.298770	1.162077	-2.201360
1	0	4.067171	1.335798	1.624207
1	0	4.083132	1.097352	5.129791
1	0	2.892203	1.416811	3.823776
1	0	2.836034	-0.117737	4.709807
1	0	5.173950	-1.611101	4.457917
1	0	6.485815	-2.112360	2.041028
1	0	-1.028350	0.145622	0.690631
1	0	-1.317397	0.877389	2.274420
1	0	-3.997266	0.120464	3.151393
1	0	-1.698565	-2.210759	0.483471
1	0	-3.737656	-3.782727	1.564925
1	0	-5.468467	-3.406034	3.336226
1	0	-5.263811	-1.997812	4.398064
1	0	-7.503037	-2.021613	3.282656
1	0	-6.708631	-1.932002	1.692232
1	0	-6.778402	0.153229	2.049782

tetramer c-a

*** imOH_NTf2_b3lyp_6-31+Gp_tetramer_D3.g09, E(RB3LYP) = -8989.00702348

0 1

6	0	5.881472	-4.643105	-1.927954
9	0	5.182952	-5.771409	-2.060346
16	0	4.926206	-3.369808	-0.919927
7	0	3.674252	-3.242221	-1.946056
16	0	2.377463	-2.384701	-1.577191
6	0	1.214989	-3.533610	-0.626371
9	0	1.729486	-3.866193	0.563897
9	0	7.023801	-4.902399	-1.277572

9	0	6.175326	-4.155609	-3.142694
8	0	5.805748	-2.174594	-0.909262
8	0	4.627244	-3.981238	0.379211
8	0	2.599973	-1.259658	-0.648201
8	0	1.625627	-2.117394	-2.814885
9	0	0.961017	-4.639792	-1.324696
9	0	0.059153	-2.867907	-0.416814
8	0	6.752466	-1.629522	1.822821
6	0	6.171207	-0.354397	2.052992
6	0	4.737932	-0.230664	1.549212
7	0	3.786811	-1.110455	2.256497
6	0	2.460384	-0.921736	2.243862
8	0	2.625173	1.837537	0.201238
16	0	2.396685	3.052550	1.015405
7	0	3.684421	3.691279	1.701101
16	0	5.152747	3.773970	1.017743
6	0	6.147295	3.714551	2.616427
9	0	5.878670	2.584875	3.292920
6	0	1.804841	4.312000	-0.259367
9	0	0.699207	3.840541	-0.854763
8	0	1.321069	2.969805	2.016771
9	0	1.547479	5.487676	0.301127
9	0	2.763461	4.456886	-1.195019
8	0	5.452236	5.059637	0.391164
8	0	5.579375	2.547728	0.296915
9	0	7.448973	3.709911	2.280694
9	0	5.894741	4.765231	3.392429
8	0	8.305351	2.226591	-1.121309
6	0	7.339059	1.914006	-2.105149
6	0	6.690167	0.550528	-1.793063
7	0	5.418104	0.377917	-2.507158
6	0	4.278934	0.986862	-2.155939
1	0	4.390485	0.792961	1.671379
1	0	4.673281	-0.480860	0.488833
1	0	6.748567	0.447965	1.573768
1	0	6.215814	-0.185655	3.133794
1	0	6.638572	-1.867233	0.882771
1	0	7.336238	-0.288625	-2.051944
1	0	6.475912	0.491494	-0.728021
1	0	7.859667	1.882978	-3.067885
1	0	6.558890	2.684650	-2.156606
1	0	7.825274	2.545628	-0.337549
7	0	3.329469	0.651757	-3.036456
1	0	4.140706	1.604478	-1.281073
6	0	1.939853	1.115061	-2.991519
6	0	5.187228	-0.378122	-3.641369
6	0	3.874909	-0.207307	-3.974579
1	0	5.955181	-0.997250	-4.077174
1	0	3.274872	-0.655400	-4.749819
1	0	1.785136	1.889507	-3.747386
1	0	1.287422	0.262293	-3.177103
1	0	1.745272	1.517070	-1.998743
6	0	4.064454	-2.286480	2.934153
7	0	1.881951	-1.927433	2.909128
6	0	2.868732	-2.792620	3.350274
1	0	1.936695	-0.116052	1.751488
6	0	0.439080	-2.113382	3.079796
1	0	0.188027	-2.119532	4.143343
1	0	-0.072503	-1.293110	2.578816
1	0	0.137697	-3.054860	2.620051
1	0	2.631157	-3.695046	3.891326
1	0	5.071955	-2.662047	3.008882
6	0	-2.457723	-5.336291	1.361077

9	0	-3.307748	-6.311537	1.038769
16	0	-3.346614	-3.679063	1.481383
7	0	-3.763568	-3.585822	-0.077117
16	0	-4.687085	-2.397161	-0.637300
6	0	-6.468451	-2.996628	-0.441849
9	0	-6.791030	-3.146854	0.850097
9	0	-1.927683	-5.593366	2.570029
9	0	-1.468339	-5.289577	0.462004
8	0	-2.274582	-2.723625	1.872863
8	0	-4.437058	-3.854912	2.447714
8	0	-4.636627	-1.154196	0.155579
8	0	-4.519673	-2.316186	-2.091524
9	0	-6.652218	-4.156666	-1.077107
9	0	-7.291136	-2.070329	-0.977383
8	0	-2.567666	-1.652007	4.544593
6	0	-2.454623	-0.253899	4.316356
6	0	-3.070905	0.200123	2.998878
7	0	-4.535986	0.042095	2.927338
6	0	-5.306568	0.766167	2.106113
8	0	-5.158961	1.982543	-0.676799
16	0	-5.584733	3.385969	-0.898889
7	0	-4.455146	4.512368	-0.773327
16	0	-2.861573	4.217664	-0.711413
6	0	-2.575867	4.251760	1.153513
9	0	-3.393463	3.348751	1.759650
6	0	-5.987391	3.429584	-2.739036
9	0	-6.885334	2.468590	-3.031107
8	0	-6.815470	3.813488	-0.228285
9	0	-6.478870	4.610775	-3.107647
9	0	-4.866825	3.178546	-3.451296
8	0	-2.136194	5.373257	-1.226408
8	0	-2.435172	2.861855	-1.102960
9	0	-1.311205	3.906314	1.430647
9	0	-2.827073	5.454228	1.667399
8	0	-0.242132	0.678550	1.378816
6	0	-0.387246	0.626387	-0.045579
6	0	-1.729961	-0.051036	-0.295530
7	0	-2.050099	-0.210798	-1.718414
6	0	-3.160976	0.268438	-2.287604
1	0	-2.844279	1.253186	2.823422
1	0	-2.646724	-0.382517	2.183216
1	0	-1.400836	0.062906	4.291313
1	0	-2.933755	0.241255	5.167693
1	0	-2.381259	-2.129816	3.711524
1	0	-1.752746	-1.039580	0.168593
1	0	-2.526602	0.555167	0.129354
1	0	0.440102	0.051850	-0.478357
1	0	-0.395791	1.629325	-0.478791
1	0	0.162048	1.533366	1.628194
7	0	-3.201963	-0.150409	-3.555821
1	0	-3.903483	0.875656	-1.788003
6	0	-4.355969	-0.014004	-4.442520
6	0	-1.361633	-0.974244	-2.648399
6	0	-2.088039	-0.936940	-3.800069
1	0	-0.434962	-1.475753	-2.422853
1	0	-1.921537	-1.409763	-4.755300
1	0	-4.007226	0.154044	-5.463698
1	0	-4.952527	-0.928297	-4.385687
1	0	-4.953299	0.837977	-4.120503
6	0	-5.306495	-0.966088	3.485815
7	0	-6.548220	0.275858	2.137822
6	0	-6.568873	-0.812415	2.993382
1	0	-4.973661	1.560317	1.457705

6	0	-7.654825	0.727524	1.288614
1	0	-8.575748	0.719817	1.875401
1	0	-7.450488	1.737931	0.933812
1	0	-7.744427	0.052075	0.434470
1	0	-7.465384	-1.387840	3.161120
1	0	-4.877259	-1.701935	4.145724

dimer c-c

*** imOH_NTf2_cation_cation_b3lyp_6-31+Gp_dimer_D3.g09, E(RB3LYP) = -4494.47756025

0 1

6	0	-2.173594	2.556073	-1.398105
9	0	-2.315883	3.860738	-1.656579
9	0	-2.457210	1.857717	-2.513745
9	0	-0.898180	2.312033	-1.062796
16	0	-3.324005	2.052375	0.012635
7	0	-3.010660	0.454123	0.127042
16	0	-3.897581	-0.682154	-0.601176
8	0	-4.644356	-0.308548	-1.797677
8	0	-4.668736	2.437018	-0.401522
8	0	-2.706245	2.679561	1.195122
8	0	-3.040563	-1.895922	-0.665196
6	0	-5.168222	-1.145712	0.715895
9	0	-5.971351	-2.108329	0.251102
9	0	-5.897138	-0.082394	1.059754
9	0	-4.527984	-1.606983	1.810097
8	0	-1.549672	-3.277475	1.212676
6	0	-1.143939	-2.329822	2.189448
6	0	-0.442365	-1.142384	1.513823
7	0	-0.003768	-0.138365	2.487400
6	0	-0.546274	1.075093	2.625863
6	0	1.071091	-0.248605	3.355416
8	0	-0.585978	-5.368063	-0.533963
6	0	-0.525660	-4.384801	-1.548175
6	0	0.639855	-3.430016	-1.243705
7	0	0.667130	-2.234250	-2.102048
6	0	-0.402698	-1.397428	-2.384876
6	0	1.775764	-1.703235	-2.630019
8	0	1.517883	0.451727	-0.497902
16	0	2.360221	1.401551	0.248169
8	0	1.728307	2.222227	1.286918
7	0	3.741981	0.816632	0.837098
16	0	4.571027	-0.393339	0.145384
8	0	5.982728	-0.263059	0.488294
8	0	4.184432	-0.744025	-1.235228
6	0	3.997435	-1.888411	1.149399
9	0	4.673338	-2.981073	0.773479
9	0	4.173629	-1.697386	2.465596
9	0	2.676578	-2.124697	0.942720
6	0	2.919568	2.679660	-1.034037
9	0	3.870285	2.169416	-1.836685
9	0	1.869780	3.034711	-1.800511
9	0	3.404145	3.767943	-0.432886
1	0	1.688742	-1.131784	3.391931
1	0	-1.371475	1.478665	2.051482
1	0	-1.121522	-0.643446	0.822029
1	0	0.440155	-1.455590	0.960217
1	0	-0.462558	-2.850509	2.870382
1	0	-2.003714	-1.970018	2.771887

1	0	-2.228224	-2.859947	0.639480
1	0	-0.929203	-4.929993	0.269581
1	0	-0.361052	-4.903822	-2.497874
1	0	-1.469290	-3.828361	-1.623695
1	0	0.554199	-3.100296	-0.209397
1	0	1.596465	-3.944439	-1.358085
1	0	2.777794	-2.080425	-2.504210
1	0	-1.397521	-1.592495	-2.014466
7	0	1.451704	-0.569844	-3.258491
6	0	0.092996	-0.358606	-3.114614
6	0	2.403093	0.321443	-3.924598
1	0	-0.396578	0.517520	-3.509236
1	0	2.073702	1.350126	-3.777240
1	0	3.381176	0.193894	-3.460010
1	0	2.446858	0.091343	-4.992605
7	0	0.134023	1.739220	3.565008
6	0	1.159397	0.934151	4.026532
6	0	-0.029401	3.167501	3.840280
1	0	0.040137	3.339607	4.916412
1	0	0.753614	3.715380	3.309856
1	0	-1.008260	3.483702	3.476155
1	0	1.874326	1.281138	4.755888

trimer c-c

*** imOH_NTf2_cation_cation_b3lyp_6-31+Gp_trimer_D3.g09, E(RB3LYP) = -6741.73332253

0 1

6	0	-4.610987	1.660311	0.233346
7	0	-3.375602	2.170411	0.170873
6	0	-3.401065	3.470341	0.646802
6	0	-4.689556	3.735617	1.005701
7	0	-5.428965	2.592289	0.737614
6	0	-2.187169	1.444861	-0.311940
6	0	-2.168930	1.333007	-1.845610
8	0	-1.047669	0.568128	-2.252792
6	0	-6.857705	2.404717	0.989274
8	0	0.077371	-1.856682	-1.424960
6	0	0.598390	-2.664680	-2.460029
6	0	-0.323668	-3.875481	-2.585916
7	0	-0.407315	-4.583075	-1.298241
6	0	0.547675	-5.366169	-0.782814
7	0	0.218875	-5.662772	0.479595
6	0	-0.985723	-5.043631	0.779490
6	0	-1.370421	-4.357140	-0.332740
6	0	1.024553	-6.481277	1.388358
8	0	1.330119	0.448177	-0.866324
6	0	1.450684	1.014041	0.429783
6	0	2.929175	1.015630	0.811332
7	0	3.721290	1.641508	-0.258362
6	0	3.781108	2.951915	-0.519703
7	0	4.440586	3.127010	-1.670691
6	0	4.810239	1.882342	-2.158986
6	0	4.347008	0.950499	-1.277499
6	0	4.726582	4.421159	-2.292924
8	0	0.436484	3.240864	-1.760969
16	0	0.524941	4.532317	-1.065951
8	0	1.817849	5.234685	-1.044845
7	0	-0.230911	4.362574	0.348990
6	0	-0.631666	5.661151	-2.037462

8	0	-5.069787	-0.718319	-2.513839
16	0	-4.475315	-1.723622	-1.629662
7	0	-5.031686	-1.458463	-0.141165
6	0	-5.262017	-3.357229	-2.152886
8	0	-3.022056	-1.979358	-1.706088
9	0	2.720665	-3.995024	2.695660
6	0	2.600266	-2.956758	1.850718
16	0	3.638456	-3.225504	0.294427
9	0	1.302512	-2.840626	1.509300
9	0	2.973503	-1.837677	2.489525
1	0	0.593622	0.869237	-1.364101
1	0	3.095822	1.562232	1.742412
1	0	3.314667	0.006608	0.923731
1	0	0.901368	0.423559	1.178020
1	0	1.053158	2.032462	0.438754
1	0	0.752879	-1.197471	-1.137089
1	0	1.623606	-2.982047	-2.228872
1	0	0.606911	-2.137706	-3.427717
1	0	-1.337746	-3.556566	-2.834601
1	0	0.035026	-4.572752	-3.348594
1	0	-1.164103	-0.377091	-2.015940
1	0	-3.103875	0.890528	-2.211142
1	0	-2.047790	2.324534	-2.288273
1	0	-1.317333	2.002255	0.035378
1	0	-2.194669	0.456246	0.153033
1	0	1.463103	-5.653407	-1.272684
1	0	-1.451465	-5.119789	1.749118
1	0	-2.210472	-3.709378	-0.511257
1	0	-2.503260	4.065230	0.706446
1	0	-5.134669	4.620839	1.432541
1	0	-4.898574	0.653471	-0.041646
1	0	4.393931	-0.126129	-1.268118
1	0	5.360874	1.775095	-3.080288
1	0	3.336846	3.740015	0.065900
1	0	-7.135187	1.391831	0.694564
1	0	-7.061030	2.538492	2.054621
1	0	-7.432328	3.128266	0.405692
1	0	4.021430	5.156813	-1.905669
1	0	5.755805	4.720415	-2.076318
1	0	4.586419	4.328050	-3.371859
1	0	0.946755	-6.062729	2.392830
1	0	0.663875	-7.513525	1.385049
1	0	2.066315	-6.432507	1.069002
9	0	-0.809431	6.820567	-1.395097
9	0	-0.119352	5.903024	-3.251427
9	0	-1.829434	5.062397	-2.184670
16	0	-0.045455	5.363186	1.627563
8	0	-1.326553	5.383798	2.341676
6	0	1.071037	4.305453	2.722814
8	0	0.704292	6.595739	1.397033
9	0	1.296900	4.921980	3.885288
9	0	0.517869	3.105576	2.963627
9	0	2.262871	4.094841	2.112086
8	0	3.288012	-2.070782	-0.553724
7	0	5.136914	-3.329122	0.841381
8	0	3.210733	-4.561974	-0.155843
16	0	6.142856	-2.054824	0.988929
8	0	7.164392	-2.385172	1.976314
6	0	7.044519	-2.083295	-0.670542
8	0	5.494385	-0.733622	1.013669
9	0	7.941673	-1.084696	-0.710477
9	0	7.677647	-3.244247	-0.862532
9	0	6.165678	-1.901201	-1.679637

9	0	-4.788940	-4.361332	-1.398439
9	0	-6.591305	-3.303302	-2.023971
9	0	-4.954586	-3.602633	-3.436795
16	0	-4.240321	-1.920038	1.208402
6	0	-5.754897	-1.916860	2.330099
8	0	-3.731517	-3.298175	1.218321
8	0	-3.383924	-0.843628	1.730707
9	0	-5.365782	-2.209267	3.577258
9	0	-6.331385	-0.697791	2.334587
9	0	-6.654264	-2.818639	1.927841

tetramer l c-c

*** imOH_NTf2_cation_cation_b3lyp_6-31+Gp_tetramer_l_D3.g09

0 1

6	0	-6.054456	-0.813523	1.217330
7	0	-5.514345	0.271217	0.651670
6	0	-5.584301	1.324879	1.551844
6	0	-6.184964	0.842442	2.675532
7	0	-6.475252	-0.492402	2.444142
6	0	-4.873502	0.320286	-0.672609
6	0	-3.347316	0.369469	-0.508256
8	0	-2.719191	0.725478	-1.740179
6	0	-6.996640	-1.444099	3.425591
8	0	-0.117409	1.240142	-1.030546
6	0	-0.123335	1.128215	0.390446
6	0	0.999453	1.979679	0.970236
7	0	0.791105	3.412428	0.762868
6	0	0.977018	4.049958	-0.396300
7	0	0.759048	5.353536	-0.203047
6	0	0.403631	5.554022	1.120164
6	0	0.432523	4.335779	1.726735
6	0	0.886817	6.403488	-1.212711
8	0	1.362024	-0.536860	-2.455727
6	0	2.620539	-0.234219	-3.058306
6	0	3.695958	-1.142885	-2.468295
7	0	4.086090	-0.771958	-1.102299
6	0	5.345572	-0.718282	-0.658232
7	0	5.334796	-0.476814	0.652875
6	0	4.021783	-0.368243	1.066965
6	0	3.237074	-0.569173	-0.029040
6	0	6.529384	-0.279146	1.476218
8	0	-0.148094	-2.385324	-3.795977
6	0	0.107450	-3.440495	-2.898991
6	0	-0.526230	-3.142095	-1.530724
7	0	-0.180944	-4.182523	-0.549399
6	0	0.680147	-4.031829	0.457079
7	0	0.792230	-5.193239	1.110853
6	0	-0.044101	-6.117335	0.504755
6	0	-0.646631	-5.483305	-0.541997
6	0	1.648224	-5.424663	2.276339
7	0	5.109782	-2.244659	-1.193441
16	0	3.924114	-3.184516	-0.673960
6	0	3.903651	-4.753791	-1.739861
9	0	4.876661	-5.599742	-1.413741
16	0	6.703060	-2.480880	-0.956190
8	0	7.154569	-3.867954	-1.071830
8	0	7.373713	-1.412000	-1.708208
6	0	7.018216	-2.007734	0.856418
9	0	6.305272	-0.909044	1.184824

8	0	2.667328	-2.517573	-1.070247
8	0	4.012299	-3.692289	0.706983
9	0	2.715596	-5.371662	-1.543138
9	0	4.001988	-4.435154	-3.039477
9	0	6.689172	-2.996777	1.688677
9	0	8.320705	-1.725044	1.007729
8	0	-4.163506	-2.792435	-1.475567
16	0	-4.426827	-3.974861	-0.638612
6	0	-6.233375	-4.416489	-0.948450
9	0	-6.366581	-4.776268	-2.227747
7	0	-4.630253	-3.595329	0.939047
16	0	-3.391432	-3.097742	1.840492
6	0	-2.976843	-4.626976	2.869887
9	0	-3.960351	-4.885345	3.741435
8	0	-3.880417	-2.133571	2.835141
8	0	-2.154021	-2.796614	1.100945
8	0	-3.678967	-5.207328	-0.918770
9	0	-7.021584	-3.342793	-0.721079
9	0	-6.634233	-5.418717	-0.162735
9	0	-2.801401	-5.696086	2.082757
9	0	-1.836463	-4.411307	3.555761
8	0	5.779566	2.346297	-0.402117
16	0	4.511256	3.070886	-0.539267
8	0	3.472224	2.502256	-1.422382
7	0	3.801688	3.618191	0.796936
16	0	4.203833	3.275126	2.322970
8	0	5.020499	2.069353	2.510028
6	0	4.940811	4.702655	-1.382010
9	0	3.818630	5.417030	-1.609163
6	0	5.329723	4.702605	2.822380
9	0	6.456076	4.671310	2.096262
8	0	2.994284	3.448475	3.137361
9	0	4.714235	5.876971	2.619216
9	0	5.643370	4.594405	4.120300
9	0	5.530080	4.453236	-2.559348
9	0	5.765584	5.431747	-0.624545
7	0	-5.054082	3.356139	-0.039589
16	0	-6.471309	3.962398	0.536209
8	0	-6.574023	3.617203	1.957664
16	0	-3.966963	4.213597	-0.842055
8	0	-3.856706	5.639295	-0.521678
8	0	-2.730918	3.400053	-0.876411
6	0	-4.524174	4.158278	-2.648393
9	0	-5.685043	4.782400	-2.829378
9	0	-3.589180	4.737855	-3.428881
9	0	-4.649059	2.870406	-3.035099
8	0	-6.850269	5.287352	0.050187
6	0	-7.641071	2.747923	-0.316378
9	0	-7.527434	2.830162	-1.649584
9	0	-7.358047	1.481137	0.054237
9	0	-8.901406	3.023822	0.033309
1	0	-1.048216	1.172893	-1.357289
1	0	1.102115	1.824122	2.046005
1	0	1.947176	1.720284	0.498529
1	0	0.051847	0.083823	0.689856
1	0	-1.091810	1.442181	0.794107
1	0	0.934509	0.237534	-2.023376
1	0	2.891813	0.818115	-2.929990
1	0	2.554097	-0.455543	-4.131743
1	0	3.334997	-2.174014	-2.437311
1	0	4.600342	-1.099500	-3.081834
1	0	0.325248	-1.600439	-3.440433
1	0	1.184364	-3.600465	-2.751263

```
      1           0        -0.321185   -4.353525   -3.327356
      1           0        -1.613305   -3.059775   -1.590628
      1           0        -0.125084   -2.203223   -1.143441
      1           0        -3.008028    1.634391   -1.948946
      1           0        -3.098433    1.103374    0.268517
      1           0        -2.959466   -0.612009   -0.226156
      1           0        -5.171754   -0.562350   -1.239690
      1           0        -5.230678    1.223156   -1.170069
      1           0         6.229788   -0.873861   -1.253583
      1           0         3.777475   -0.157024    2.094240
      1           0         2.173123   -0.648902   -0.147756
      1           0        -1.403258   -5.810482   -1.234003
      1           0        -0.138880   -7.126796    0.872470
      1           0         1.240926   -3.136787    0.656096
      1           0        -6.089278   -1.802264    0.790436
      1           0        -6.447768    1.333413    3.599520
      1           0        -5.243480    2.313973    1.288475
      1           0         0.281344    4.051135    2.754935
      1           0         0.148443    6.530951    1.496703
      1           0         1.310188    3.580640   -1.305709
      1           0         2.426377   -4.659843    2.287693
      1           0         1.046501   -5.387414    3.188683
      1           0         2.121626   -6.403720    2.176590
      1           0        -6.207940   -1.685059    4.141047
      1           0        -7.861724   -1.005233    3.927381
      1           0        -7.295257   -2.355980    2.906679
      1           0         1.079582    5.939025   -2.179770
      1           0        -0.046433    6.966300   -1.252579
      1           0         1.722699    7.056659   -0.950952
      1           0         6.871879    0.749339    1.358773
      1           0         6.261380   -0.457471    2.516485
      1           0         7.283987   -1.002779    1.164047
```

tetramer c-c

```
*** imOH_NTf2_cation_cation_b3lyp_6-31+Gp_tetramer_D3.g09,  E(RB3LYP) =  -
8989.00958615

0 1
      6           0         5.592926    2.152365   -1.690792
      7           0         5.518031    1.184931   -0.701536
      6           0         6.454162    0.255950   -0.931576
      7           0         7.136199    0.601868   -2.028967
      6           0         6.611790    1.787266   -2.520015
      6           0         4.511795    1.131098    0.372324
      6           0         3.190695    0.569226   -0.167284
      8           0         2.219919    0.563178    0.868837
      6           0         8.193999   -0.193635   -2.652550
      8           0        -0.300237    1.409752    0.425408
      6           0        -0.511866    1.104705   -0.948599
      6           0        -1.767282    1.833008   -1.405338
      7           0        -1.590926    3.284461   -1.357035
      6           0        -1.584093    4.007822   -0.233792
      7           0        -1.404510    5.291285   -0.556894
      6           0        -1.284893    5.390744   -1.933159
      6           0        -1.409862    4.130687   -2.433652
      6           0        -1.324948    6.410790    0.381073
      8           0        -1.245509   -0.506034    2.005398
      6           0        -2.273377   -0.346953    2.981836
      6           0        -3.397228   -1.340115    2.704348
      7           0        -4.175554   -1.000895    1.507739
```

6	0	-5.510508	-0.975968	1.449433
7	0	-5.885755	-0.745008	0.191120
6	0	-4.751796	-0.620113	-0.588337
6	0	-3.678765	-0.795894	0.232752
6	0	-7.273421	-0.596173	-0.252097
8	0	1.211542	-1.632833	2.210797
6	0	1.174721	-2.962549	1.720722
6	0	1.173373	-2.942665	0.183984
7	0	1.134935	-4.304499	-0.375260
6	0	2.072601	-5.299314	-0.154344
6	0	1.626983	-6.412334	-0.803946
7	0	0.427052	-6.080287	-1.412898
6	0	0.156706	-4.798344	-1.141019
6	0	-0.421835	-6.977072	-2.201744
6	0	-5.125884	4.476554	1.774886
16	0	-4.889591	2.795392	0.953589
8	0	-6.148466	2.073377	1.171833
8	0	-3.662656	2.280257	1.591824
7	0	-4.524483	3.248688	-0.544821
6	0	6.545569	-2.689634	2.315157
16	0	5.091878	-2.980935	1.152920
8	0	4.162983	-1.866385	1.401926
8	0	4.606494	-4.341933	1.435953
7	0	5.944178	-2.807809	-0.223220
8	0	1.618950	5.861806	-0.696497
16	0	2.221473	4.591479	-0.275579
8	0	1.621382	3.335330	-0.751252
7	0	3.810403	4.446302	-0.445521
6	0	1.929171	4.537142	1.593103
8	0	-2.945540	-5.352082	-0.874679
16	0	-3.009050	-4.461181	0.298172
6	0	-2.471701	-5.558060	1.743408
7	0	-4.428403	-3.840726	0.684810
8	0	-2.022783	-3.362973	0.365464
1	0	0.661175	1.267692	0.621635
1	0	-2.040508	1.570189	-2.428923
1	0	-2.607965	1.588369	-0.755263
1	0	-0.660616	0.022046	-1.092846
1	0	0.352500	1.421783	-1.540497
1	0	-1.042285	0.334599	1.521846
1	0	-2.655856	0.677451	2.978165
1	0	-1.861452	-0.571294	3.974536
1	0	-2.987067	-2.341332	2.558323
1	0	-4.095479	-1.364555	3.545703
1	0	0.294275	-1.265965	2.216895
1	0	0.286134	-3.495015	2.082373
1	0	2.070257	-3.469251	2.088817
1	0	2.072831	-2.453605	-0.189083
1	0	0.285637	-2.420353	-0.176290
1	0	2.176728	-0.302320	1.339940
1	0	2.819872	1.226141	-0.958299
1	0	3.348897	-0.436194	-0.573883
1	0	4.897383	0.494209	1.169030
1	0	4.366704	2.144284	0.745547
1	0	-6.179775	-1.141816	2.277133
1	0	-4.819831	-0.409221	-1.642236
1	0	-2.624343	-0.848822	0.038416
1	0	2.967725	-5.119410	0.422305
1	0	2.055366	-7.398760	-0.889438
1	0	-0.727933	-4.267944	-1.447997
1	0	6.590819	-0.656408	-0.369186
1	0	7.018189	2.274746	-3.392406
1	0	4.944621	3.016420	-1.675864

1	0	-1.410150	3.765722	-3.447700
1	0	-1.104214	6.335963	-2.418903
1	0	-1.723606	3.607749	0.756086
1	0	-1.419420	-6.542899	-2.269983
1	0	0.009433	-7.117679	-3.196496
1	0	-0.490902	-7.937477	-1.686680
1	0	7.811493	-0.649084	-3.567862
1	0	9.048803	0.452028	-2.866556
1	0	8.489582	-0.987068	-1.966197
1	0	-1.471776	6.031805	1.391721
1	0	-0.337595	6.867962	0.297082
1	0	-2.111781	7.131634	0.146574
1	0	-7.613433	0.412363	-0.014664
1	0	-7.305865	-0.743799	-1.330132
1	0	-7.875387	-1.360570	0.242582
9	0	2.555080	3.487351	2.142017
9	0	2.361978	5.660502	2.175507
9	0	0.604877	4.415601	1.841310
16	0	4.890318	5.675136	-0.405322
6	0	5.908674	5.124746	1.088848
8	0	5.811996	5.497112	-1.532921
8	0	4.370037	6.996693	-0.059185
9	0	6.933468	5.966742	1.269235
9	0	5.160295	5.104990	2.201399
9	0	6.404214	3.883509	0.892572
9	0	-2.492796	-4.854962	2.889970
9	0	-3.240746	-6.634743	1.875584
9	0	-1.199063	-5.956669	1.517410
16	0	-5.874092	-4.582140	0.529141
6	0	-6.300368	-4.428478	-1.313234
8	0	-5.878684	-6.019190	0.801436
8	0	-6.845224	-3.691555	1.176997
9	0	-7.600847	-4.709483	-1.475798
9	0	-6.079965	-3.169004	-1.744357
9	0	-5.576195	-5.265731	-2.058976
9	0	7.503720	-3.599801	2.131305
9	0	6.099148	-2.762282	3.572590
9	0	7.058894	-1.461817	2.105775
16	0	5.259755	-3.083510	-1.659395
8	0	5.952772	-2.263805	-2.659676
6	0	5.774180	-4.852244	-2.070231
8	0	3.787092	-3.123748	-1.644518
9	0	5.287749	-5.187676	-3.275557
9	0	7.108034	-4.959278	-2.093446
9	0	5.284425	-5.703913	-1.156461
9	0	-6.146096	5.140842	1.222707
9	0	-4.007320	5.220201	1.645049
9	0	-5.370054	4.303209	3.080832
16	0	-5.293253	2.868570	-1.911598
8	0	-4.300406	2.918275	-2.992177
8	0	-6.206347	1.720567	-1.836013
6	0	-6.407231	4.358074	-2.218976
9	0	-6.989011	4.245062	-3.420338
9	0	-7.358397	4.423323	-1.277418
9	0	-5.688125	5.491170	-2.188641

[HEPy][NTf_2]

monomer c-a

```
*** pyridinium_NTf2_b3lyp_6-31+Gp_monomer_D3, E(RB3LYP) =  -2229.95481608

0 1
 C    -0.493892    -2.848177    -0.266739
 F     0.193411    -3.167655    -1.366247
 S    -0.184199    -1.062391     0.237794
 O    -0.577604    -0.260266    -0.941528
 O    -1.005732    -0.855316     1.450497
 N     1.394547    -1.205511     0.499891
 S     2.289476     0.057381     0.954094
 O     3.427938    -0.390852     1.745368
 F    -1.813425    -2.961521    -0.536296
 F    -0.185753    -3.696232     0.715763
 O     1.490670     1.220332     1.412036
 C     3.044023     0.656078    -0.670285
 F     3.716810    -0.327027    -1.276352
 F     2.078722     1.096224    -1.498632
 F     3.883818     1.672923    -0.419728
 H     1.122575     3.044404     1.019332
 O     0.512805     3.724212     0.669473
 C    -0.454472     3.053008    -0.107178
 C    -1.456537     2.384873     0.847099
 H    -0.940379     3.819274    -0.722551
 H    -0.005508     2.295097    -0.760581
 N    -2.532707     1.577770     0.187455
 H    -0.912288     1.689876     1.489758
 H    -1.948488     3.143770     1.462706
 C    -3.230772     0.716538     0.962438
 C    -4.202947    -0.106048     0.418801
 C    -4.450752    -0.052712    -0.953026
 C    -3.716066     0.834161    -1.739982
 C    -2.754582     1.633529    -1.143436
 H    -2.965612     0.697156     2.010632
 H    -4.730676    -0.795462     1.067871
 H    -5.194616    -0.702646    -1.403613
 H    -3.859588     0.896943    -2.812672
 H    -2.137522     2.309169    -1.716304
```

dimer c-a

```
*** pyridinium_NTf2_b3lyp_6-31+Gp_dimer_D3, E(RB3LYP) =  -4459.97150546

0 1
 O    -2.937008    -0.066850    -2.067654
 S    -3.644977    -0.617525    -0.881644
 C    -5.470526    -0.386107    -1.283367
 F    -5.743179    -1.098704    -2.384513
 N    -3.621429     0.369691     0.399487
 S    -2.349178     0.456527     1.380459
 O    -1.073752     0.039227     0.767765
 C    -2.661432    -0.827320     2.728800
 F    -2.643881    -2.064712     2.215147
 F    -1.687271    -0.729674     3.648791
 F    -3.842382    -0.614088     3.317390
 O    -2.394532     1.736455     2.095216
 O    -3.442224    -2.046962    -0.611219
```

F	-5.739081	0.906209	-1.525590
F	-6.236735	-0.816280	-0.279615
C	0.727209	-2.132074	0.382644
N	0.028753	-2.315913	-0.762373
C	-0.802331	-3.374495	-0.909983
C	-0.927282	-4.313255	0.101864
C	-0.202208	-4.151925	1.282488
C	0.147052	-1.260615	-1.813574
C	-0.105779	-1.730776	-3.241475
O	-1.448873	-2.106142	-3.517328
C	-0.478319	2.769104	-0.499621
N	-0.495170	3.627617	0.547730
C	-1.615120	4.319579	0.857541
C	-2.765184	4.186388	0.099526
C	-2.764976	3.312430	-0.988876
C	0.714115	3.728050	1.406616
C	0.841813	2.532121	2.374214
O	2.039720	2.670761	3.109394
O	3.678629	2.441825	0.706187
S	3.398630	1.331943	-0.222689
N	4.619320	0.311973	-0.236771
S	4.499390	-1.277020	-0.576558
O	5.768933	-1.743729	-1.117931
C	3.470059	2.142154	-1.921973
F	4.630720	2.760386	-2.129198
O	2.025084	0.776435	-0.184792
F	3.278927	1.220390	-2.877819
F	2.470273	3.053247	-2.008811
O	3.234723	-1.700935	-1.200263
C	4.414164	-1.985335	1.173209
F	4.181837	-3.307277	1.116531
F	5.546108	-1.768498	1.841902
F	3.393959	-1.409817	1.852200
H	-3.663380	3.160130	-1.578001
H	-0.294934	-4.873759	2.088540
H	1.159379	-0.866794	-1.734523
H	-0.558158	-0.477559	-1.529879
H	0.220098	-0.909905	-3.898442
H	0.515654	-2.601430	-3.476230
H	-2.049139	-1.395987	-3.216517
H	1.342268	-1.245811	0.424999
H	-1.607483	-5.145356	-0.040297
H	-1.361378	-3.417473	-1.835793
H	0.657673	4.664887	1.964132
H	1.585764	3.764647	0.750773
H	0.012200	2.533855	3.085670
H	0.803132	1.594524	1.808113
H	2.787614	2.509078	2.501936
H	0.438073	2.209712	-0.644178
H	-1.557045	4.957689	1.730711
H	-3.652908	4.739196	0.384828
C	-1.601290	2.605973	-1.292098
H	-1.580551	1.888449	-2.101451
C	0.633149	-3.043298	1.419856
H	1.213012	-2.869394	2.318535

trimer c-a

*** pyridinium_NTf2_b3lyp_6-31+Gp_trimer_D3, E(RB3LYP) = -6689.96905432

0 1

C	1.372374	3.084930	3.760229

C	0.626173	2.079877	3.167501
N	-0.696441	2.251110	2.930082
C	-1.319035	3.408602	3.251688
C	-0.615596	4.436689	3.859667
C	0.747195	4.281592	4.114494
C	-1.408648	1.149041	2.210391
C	-2.915105	1.082173	2.423180
O	-3.650586	2.186520	1.902564
O	-2.726811	2.951900	-0.616902
S	-1.430870	3.657421	-0.801128
O	-0.691900	3.974715	0.430012
C	-1.915924	5.300539	-1.580621
F	-0.836284	6.031627	-1.858155
N	-0.590188	3.088545	-2.049064
S	0.424262	1.844269	-1.878496
O	0.424214	1.045351	-3.111366
F	-2.683592	5.962740	-0.701031
F	-2.621743	5.101834	-2.703895
O	0.289168	1.127091	-0.594255
C	2.126926	2.653168	-1.814039
F	2.359202	3.317263	-2.952129
F	2.175350	3.510813	-0.786755
F	3.048025	1.705083	-1.650390
F	3.162801	1.748442	1.520410
C	4.460590	1.572248	1.844389
F	5.213373	2.324092	1.043211
F	4.622970	1.996575	3.116437
S	4.934952	-0.249559	1.753720
O	6.362918	-0.316704	2.064975
O	4.001809	-0.904324	2.702516
N	4.518363	-0.416259	0.202271
S	4.854061	-1.716686	-0.686227
O	4.113129	-1.584241	-1.945429
C	6.668441	-1.560332	-1.199890
F	7.482929	-1.790117	-0.168526
O	4.780419	-2.993603	0.048825
F	6.906804	-0.335120	-1.687746
F	6.918140	-2.466179	-2.161635
C	-4.841453	1.765165	-2.485248
N	-4.661503	0.880176	-1.477996
C	-3.548079	0.111271	-1.421348
C	-2.572684	0.211070	-2.396047
C	-2.732065	1.126498	-3.435351
C	-3.890273	1.906844	-3.480244
C	-5.691993	0.695895	-0.424680
C	-6.516361	-0.585044	-0.676851
O	-7.244676	-0.924234	0.481285
O	-4.365743	-1.694553	1.105806
S	-4.067006	-3.034773	1.669575
C	-3.049232	-2.657103	3.215938
F	-1.879641	-2.081983	2.871559
N	-3.058913	-3.956239	0.813772
S	-1.996388	-3.405444	-0.258453
O	-1.716582	-1.954116	-0.230767
O	-0.858799	-4.323756	-0.335512
C	-2.899586	-3.653150	-1.896798
F	-2.148011	-3.151958	-2.897682
O	-5.194237	-3.836227	2.133363
F	-3.133997	-4.942315	-2.135017
F	-4.074233	-2.989570	-1.885603
F	-3.725632	-1.799142	3.999768
F	-2.797014	-3.771351	3.900871
O	1.243092	-0.985994	2.644846

```
 C     1.196682    -1.142354     1.230444
 C     1.597815    -2.570003     0.863515
 N     1.627038    -2.822187    -0.606659
 C     2.133520    -4.004919    -1.023959
 C     2.164465    -4.329777    -2.365901
 C     1.677471    -3.413398    -3.300638
 C     1.173438    -2.195483    -2.855312
 C     1.156403    -1.924527    -1.496681
 H     1.706350    -3.643550    -4.361382
 H     1.315979     5.082469     4.577577
 H    -1.690102    -0.407760    -2.307792
 H    -0.962042     0.217213     2.561221
 H    -1.161071     1.266836     1.152917
 H    -3.240616     0.140813     1.965675
 H    -3.156639     1.025651     3.490094
 H    -3.335712     2.407072     1.000834
 H    -6.324778     1.585041    -0.404691
 H    -5.176560     0.628556     0.533006
 H    -7.242546    -0.415101    -1.479592
 H    -5.843986    -1.395067    -0.983668
 H    -6.634887    -1.382786     1.086778
 H    -3.469005    -0.569894    -0.583616
 H    -5.750317     2.353795    -2.449574
 H    -1.949395     1.243465    -4.176620
 H     1.037274     1.114629     2.886494
 H    -1.139479     5.353390     4.106650
 H    -2.367451     3.473775     2.988905
 H     2.603810    -2.780507     1.234999
 H     0.887445    -3.282173     1.290677
 H     1.853989    -0.419138     0.737543
 H     0.169045    -0.950136     0.924735
 H     2.192228    -0.997896     2.891857
 H     2.510782    -4.659529    -0.249687
 H     0.776961    -0.991102    -1.112791
 H     0.814249    -1.429582    -3.532660
 H    -4.047530     2.642130    -4.261298
 H     2.580636    -5.284498    -2.666687
 H     2.429602     2.917932     3.929515
```

tetramer c-a

*** pyridinium_NTf2_b3lyp_6-31+Gp_tetramer_D3.g09, E(RB3LYP) = -8919.71359722

```
0 1
 C     4.026895    -0.377175     3.319361
 N     3.666395    -1.130768     2.255053
 C     2.479322    -1.785331     2.233561
 C     1.620152    -1.712339     3.316828
 C     1.972029    -0.929479     4.415840
 C     4.608701    -1.287981     1.120730
 C     5.374116    -2.622928     1.195697
 O     6.392174    -2.640708     0.232158
 O     5.156779     1.920489     1.640465
 S     4.426249     3.094966     2.191365
 C     5.342319     3.478353     3.773044
 F     6.593262     3.809164     3.452764
 N     3.015208     2.733086     2.907028
 S     1.757346     2.196068     2.060181
 O     2.114658     1.531679     0.793984
 C     0.836963     3.747230     1.520392
 F     1.679260     4.656011     1.031049
```

F	-0.045523	3.419317	0.557527
F	0.170753	4.270598	2.552394
O	0.825110	1.518703	2.966599
O	4.437025	4.300667	1.354925
F	5.368916	2.382520	4.553384
F	4.769158	4.477380	4.434182
C	1.940113	1.554077	-2.232559
N	3.113917	2.077587	-1.811305
C	3.264964	3.414231	-1.651099
C	2.218588	4.271442	-1.946769
C	1.001275	3.751629	-2.388202
C	4.182843	1.143955	-1.359901
C	5.601544	1.526022	-1.770507
O	6.096382	2.679136	-1.106023
F	-2.071200	5.304254	-1.989717
C	-2.803191	4.574888	-2.838334
F	-2.021383	4.212044	-3.869345
S	-3.384867	3.004403	-2.017304
O	-2.146547	2.298576	-1.622771
N	-4.177218	3.684048	-0.790979
S	-5.010334	2.755625	0.241757
C	-6.772907	2.891587	-0.376632
F	-7.200415	4.147575	-0.312421
O	-4.695415	1.315216	0.129656
O	-5.018767	3.384033	1.561580
O	-4.224868	2.320902	-3.028252
F	-3.823463	5.293502	-3.287778
F	-7.561747	2.111261	0.376965
F	-6.848118	2.463422	-1.648831
C	-4.765599	-2.486365	-1.207242
N	-4.717462	-1.307270	-1.872467
C	-5.754912	-0.438355	-1.823810
C	-6.895854	-0.752357	-1.105599
C	-6.960734	-1.960090	-0.413346
C	-3.501738	-0.937212	-2.643511
C	-3.742253	-0.957856	-4.156869
O	-4.742440	-0.052048	-4.570391
C	-1.737265	-0.404265	2.704962
N	-1.858888	0.358434	1.598786
C	-2.110479	1.682962	1.684784
C	-2.280786	2.289764	2.915063
C	-2.184769	1.516126	4.070493
C	-1.762394	-0.266827	0.256712
C	-0.398132	-0.034106	-0.400700
O	-0.478907	-0.401066	-1.771148
O	0.761443	-2.861084	-2.588657
S	2.162804	-2.700411	-2.170646
N	3.099120	-2.753473	-3.463449
S	4.669112	-2.336682	-3.462758
O	5.473032	-3.325842	-4.168519
C	2.605704	-4.262748	-1.234511
F	2.187826	-5.340649	-1.879726
O	2.419645	-1.620536	-1.186626
F	3.941470	-4.319184	-1.085896
F	2.049123	-4.222797	-0.013359
O	5.171506	-1.791261	-2.177965
C	4.611174	-0.837075	-4.584419
F	5.797941	-0.220212	-4.547796
F	4.329901	-1.191104	-5.832216
F	3.672190	0.030592	-4.153317
F	-1.499607	-3.206068	-0.950236
C	-1.394592	-4.481054	-0.516087
F	-2.579932	-5.073723	-0.735465

```
 F    -0.458119    -5.104137    -1.216907
 S    -0.951836    -4.505006     1.332724
 O    -0.002979    -5.599694     1.492941
 O    -0.480291    -3.119106     1.577736
 N    -2.337109    -4.919776     2.052663
 S    -3.551097    -3.879596     2.242380
 C    -3.339079    -3.342794     4.028719
 F    -4.097886    -2.265838     4.268446
 O    -4.836118    -4.580819     2.208626
 O    -3.430050    -2.624611     1.468901
 F    -3.674255    -4.317950     4.865143
 F    -2.049052    -3.000948     4.257115
 H    -7.835672    -2.208106     0.179184
 H    -2.331244     1.969571     5.045642
 H     0.161149     4.406184    -2.587260
 H     0.687676    -2.258972     3.262123
 H     3.933227     0.162446    -1.747612
 H     4.100369     1.115960    -0.275107
 H     6.222270     0.638742    -1.578568
 H     5.652241     1.729282    -2.843934
 H     6.058258     2.505675    -0.146922
 H     5.310255    -0.456165     1.155897
 H     4.029614    -1.237352     0.200542
 H     5.855269    -2.716189     2.176627
 H     4.664313    -3.457712     1.090210
 H     5.988086    -2.597151    -0.656551
 H     2.245847    -2.344241     1.336812
 H     4.969377     0.144708     3.238675
 H     1.294128    -0.831611     5.257096
 H     1.870323     0.475900    -2.266297
 H     2.360161     5.334818    -1.793254
 H     4.226809     3.737728    -1.272054
 H    -2.703673    -1.623729    -2.365614
 H    -3.203308     0.061772    -2.329496
 H    -2.768233    -0.758502    -4.629897
 H    -4.070321    -1.953127    -4.475157
 H    -4.445413     0.848904    -4.343377
 H    -2.538589     0.199197    -0.342847
 H    -1.973499    -1.324263     0.377341
 H    -0.148494     1.027009    -0.353638
 H     0.389617    -0.585724     0.121940
 H    -0.134160    -1.306562    -1.888700
 H    -1.532239    -1.449592     2.531190
 H    -2.172071     2.212093     0.745296
 H    -2.511112     3.346641     2.946142
 H    -3.886289    -3.109938    -1.264946
 H    -7.694873    -0.023434    -1.061743
 H    -5.624738     0.493033    -2.349160
 C     3.187956    -0.248908     4.410776
 H     3.485494     0.408873     5.217944
 C    -1.904096     0.155079     3.959563
 H    -1.835495    -0.489968     4.826722
 C    -5.876448    -2.837121    -0.462565
 H    -5.854709    -3.758197     0.108359
 C     0.866005     2.372026    -2.535465
 H    -0.071629     1.910757    -2.817845
```

dimer c-c

*** pyridinium_NTf2_cation_cation_b3lyp_6-31+Gp_dimer_D3, E(RB3LYP) = -4459.95830847

0 1

C	3.041374	-0.052375	-2.832858
N	1.729000	-0.378284	-2.856479
C	1.295983	-1.487152	-3.502743
C	2.187389	-2.291187	-4.192876
C	3.545181	-1.958346	-4.198309
C	3.974441	-0.828196	-3.503172
C	0.765379	0.387062	-2.024623
C	0.714921	-0.224647	-0.621161
O	0.385324	-1.602215	-0.672769
O	-2.101275	-2.775075	-0.343941
C	-2.335341	-3.532749	0.831239
C	-1.105056	-3.467873	1.731845
N	-0.711243	-2.093242	2.163539
C	0.611068	-1.804014	2.261630
C	1.039646	-0.553130	2.663999
C	0.092579	0.441230	2.923328
C	-1.263547	0.131936	2.806842
C	-1.643356	-1.150915	2.443699
O	-2.278252	-0.034166	-1.334969
S	-2.809201	1.053739	-0.491805
O	-1.852089	1.955263	0.156632
N	-3.886892	0.605659	0.624914
S	-4.811758	-0.715023	0.553548
O	-4.650123	-1.572124	-0.640594
C	-6.554273	-0.005882	0.430233
F	-6.806121	0.778789	1.482010
F	-6.685432	0.708092	-0.693557
F	-7.431507	-1.017170	0.413323
O	-4.782958	-1.378435	1.868987
C	-3.747821	2.150389	-1.706594
F	-4.406966	3.118077	-1.061662
F	-2.863901	2.709095	-2.551069
F	-4.618616	1.420337	-2.415044
F	3.553607	2.775934	-0.897301
C	2.916242	2.969187	0.279478
F	2.928237	4.274119	0.560770
S	3.749300	1.985844	1.661126
N	3.731999	0.489191	1.044424
S	5.065471	-0.234200	0.459779
C	4.323023	-1.972650	0.449483
F	3.881085	-2.335772	1.668963
O	2.759442	2.047855	2.745026
O	5.064726	2.604703	1.812218
F	1.635181	2.574995	0.117803
O	5.307469	0.049546	-0.968126
O	6.208858	-0.310754	1.367049
F	3.287157	-2.065195	-0.409660
F	5.265951	-2.842905	0.061986
H	-2.039684	0.872424	2.959376
H	-2.937494	-2.364770	-0.651661
H	-1.272914	-4.067596	2.632948
H	-0.243993	-3.869221	1.193928
H	-2.480925	-4.596208	0.588803
H	-3.237841	-3.192039	1.349005
H	-0.586947	-1.733969	-0.683069
H	1.694757	-0.139950	-0.148117
H	0.000567	0.362050	-0.039653
H	-0.214146	0.333698	-2.500541
H	1.093217	1.426531	-1.986396
H	3.323462	0.810703	-2.244024
H	4.260577	-2.582080	-4.725795
H	1.815548	-3.170588	-4.706954

```
 H     2.100251    -0.341801     2.709972
 H     0.428414     1.440431     3.179046
 H    -2.685156    -1.429752     2.357472
 H     1.302007    -2.588566     1.983300
 H     5.018945    -0.550586    -3.432376
 H     0.236253    -1.694206    -3.434217
```

Trimer c-c

*** pyridinium_NTf2_cation_cation_b3lyp_6-31+Gp_trimer_D3, E(RB3LYP) = -6689.76328650

```
0 1
 C    -3.899720     1.392472    -1.997415
 N    -3.135828     0.413841    -2.533471
 C    -3.688901    -0.584631    -3.257763
 C    -5.053569    -0.624805    -3.480752
 C    -5.859718     0.371364    -2.935261
 C    -5.270590     1.390187    -2.187371
 C    -1.671881     0.395401    -2.287404
 C    -0.875422     0.675455    -3.561383
 O    -0.919455    -0.391553    -4.492466
 O     0.835537    -1.938488    -2.974670
 C     2.181129    -2.351578    -2.805497
 C     3.146630    -1.272253    -3.307398
 N     2.785211     0.092684    -2.839792
 C     2.458765     0.292679    -1.544350
 C     2.128088     1.551938    -1.076656
 C     2.137821     2.628919    -1.960222
 C     2.505192     2.410868    -3.288697
 C     2.824672     1.131292    -3.704197
 O    -0.544472    -2.406299    -0.674943
 C    -0.051668    -2.417372     0.666583
 C     0.181849    -0.962736     1.090149
 N     0.771765    -0.897514     2.447049
 C    -0.017774    -1.200378     3.505978
 C     0.499178    -1.172870     4.787331
 C     1.841992    -0.830932     4.978875
 C     2.626710    -0.506761     3.876245
 C     2.060014    -0.534268     2.612738
 O    -2.871022    -3.771936    -0.356873
 S    -4.154652    -3.120946    -0.714955
 C    -5.405914    -3.924606     0.422786
 F    -5.479924    -5.229184     0.154195
 O    -4.670965    -3.313196    -2.071495
 N    -4.219205    -1.546041    -0.385075
 S    -3.531993    -0.832847     0.891944
 C    -5.031042    -0.109378     1.740456
 F    -4.635521     0.530262     2.844685
 O    -2.896504    -1.715190     1.888446
 O    -2.777724     0.342236     0.434716
 F    -5.045470    -3.751701     1.696264
 F    -6.601171    -3.361681     0.225290
 F    -5.641595     0.756718     0.925118
 F    -5.886011    -1.080719     2.072552
 F    -1.697651     1.075099     2.887515
 C    -1.748542     2.406206     2.695004
 F    -1.768567     2.992391     3.896105
 S    -0.202621     2.994662     1.772959
 N    -0.461505     2.806181     0.184485
 S    -1.239889     3.871133    -0.759726
 C    -0.361893     5.543670    -0.628643
```

```
 F    -0.869594     6.288185     0.341671
 O     0.817676     1.993629     2.133245
 O    -0.045886     4.386843     2.203321
 F    -2.859283     2.713977     2.035540
 O    -0.958314     3.439514    -2.141645
 O    -2.637071     4.146075    -0.392025
 F     0.953901     5.359252    -0.416494
 F    -0.517348     6.176433    -1.797540
 O     5.407185     1.096000    -1.417848
 S     5.366723     0.330339    -0.167883
 O     4.451771     0.757390     0.910812
 N     5.226744    -1.240837    -0.549504
 S     4.535516    -2.262031     0.501718
 O     5.067932    -2.289668     1.867994
 C     5.074310    -3.838416    -0.342356
 F     6.398205    -3.952850    -0.329221
 F     4.639808    -3.854756    -1.614813
 F     4.529125    -4.867230     0.310726
 O     3.052314    -2.273344     0.370208
 C     7.083624     0.480490     0.561010
 F     7.989791     0.079442    -0.332863
 F     7.308397     1.759137     0.875987
 F     7.179131    -0.271136     1.657915
 H    -0.149362    -1.413807     5.621676
 H     1.869392     1.687914    -0.034487
 H     0.333241    -2.190796    -2.163634
 H     4.159334    -1.484162    -2.950793
 H     3.141105    -1.231564    -4.398173
 H     2.399701    -3.260679    -3.384174
 H     2.382098    -2.580614    -1.757510
 H    -0.297909    -1.069951    -4.156180
 H    -1.266233     1.572746    -4.048912
 H     0.147883     0.901113    -3.243509
 H    -1.427416    -0.582732    -1.871855
 H    -1.453865     1.137748    -1.527764
 H    -1.372799    -2.939187    -0.692757
 H     0.887086    -2.974670     0.718709
 H    -0.795396    -2.883324     1.318080
 H    -0.743668    -0.393370     1.094060
 H     0.873078    -0.476199     0.411193
 H    -3.398012     2.150080    -1.411885
 H    -6.935508     0.346020    -3.077853
 H    -5.466535    -1.459122    -4.032737
 H    -1.049858    -1.439641     3.274728
 H     2.263342    -0.810178     5.979082
 H     2.619721    -0.260435     1.732824
 H     2.530758     3.220592    -4.008459
 H     1.859093     3.616007    -1.616517
 H     2.504448    -0.577581    -0.904989
 H     3.670626    -0.230337     3.961055
 H     3.101672     0.897729    -4.724279
 H    -5.857752     2.178633    -1.731701
 H    -3.001941    -1.324014    -3.642652
```

tetramer c-c

```
*** pyridinium_NTf2_cation_cation_b3lyp_6-31+Gp_tetramer_D3.g09, E(RB3LYP) =
-8919.69071429

0 1
 C     5.645898     0.566823     0.847656
 N     4.694708     1.503249     0.637151
```

C	4.868780	2.494159	-0.272940
C	6.032307	2.558252	-1.016234
C	7.025957	1.594556	-0.821243
C	6.828920	0.593643	0.126725
C	3.392128	1.389884	1.341824
C	2.340537	0.675130	0.477665
O	1.239193	0.387754	1.330919
O	0.421957	-1.959186	2.305760
C	0.344592	-3.003481	1.348530
C	1.488874	-2.867623	0.334476
N	1.600200	-4.058564	-0.537308
C	2.130357	-5.194203	-0.023207
C	2.246203	-6.325610	-0.805515
C	1.818686	-6.283091	-2.135633
C	1.299853	-5.097769	-2.648733
C	1.213066	-3.984815	-1.827678
O	-1.531406	-0.245053	3.100397
C	-2.267600	-0.656597	4.232188
C	-3.741713	-0.923246	3.895935
N	-3.900551	-2.073160	2.978499
C	-4.485542	-3.224871	3.393272
C	-4.630050	-4.291690	2.531149
C	-4.156550	-4.177457	1.218667
C	-3.556106	-2.993442	0.815306
C	-3.468015	-1.935966	1.705890
O	-1.092162	1.785517	1.177783
C	-1.208161	2.971085	1.952859
C	-0.830995	4.154912	1.084091
N	-1.837137	4.549744	0.051304
C	-1.672472	5.764142	-0.531176
C	-2.532762	6.185391	-1.526741
C	-3.566035	5.343331	-1.941926
C	-3.707198	4.094431	-1.343227
C	-2.823877	3.714672	-0.343763
O	0.200702	-0.995857	-1.930873
S	-1.175127	-0.842035	-1.425002
O	-1.341723	-0.732950	0.035638
N	-2.284899	-1.861971	-1.966340
S	-2.153908	-2.865939	-3.232143
O	-0.817037	-2.966748	-3.835916
C	-2.343767	-4.491009	-2.304847
F	-3.607519	-4.689240	-1.909587
F	-1.561272	-4.506855	-1.199074
F	-1.966549	-5.505012	-3.090063
O	-3.333642	-2.783851	-4.085693
C	-1.746762	0.792931	-2.120530
F	-0.902077	1.760022	-1.750229
F	-2.972226	1.078117	-1.660007
F	-1.784379	0.717545	-3.450602
O	1.135049	6.577955	0.589660
S	2.442466	5.910682	0.445900
O	3.606634	6.721921	0.085883
N	2.398636	4.459349	-0.292967
S	1.791572	4.212538	-1.775428
O	1.997582	2.791209	-2.070140
O	0.481830	4.831225	-2.014069
C	2.972775	5.115205	-2.916584
F	2.843935	6.432877	-2.760190
F	4.236605	4.759496	-2.623576
F	2.713032	4.789000	-4.182227
C	2.815186	5.226874	2.146082
F	1.846326	4.379217	2.547372
F	3.979818	4.560353	2.145595

F	2.888181	6.234028	3.012735
O	-6.924091	-2.433147	0.810643
S	-6.781837	-1.108732	0.197427
C	-6.013182	-1.435218	-1.502032
F	-5.321124	-2.582220	-1.470325
O	-7.953818	-0.271613	-0.061248
N	-5.577445	-0.361870	1.025843
S	-5.300354	1.214286	0.983045
O	-5.618651	1.943208	-0.250566
C	-6.433026	1.940260	2.287650
F	-7.711630	1.792671	1.962563
O	-3.960524	1.420195	1.586624
F	-6.207147	1.329394	3.466648
F	-6.155784	3.244614	2.426802
F	-6.993987	-1.548537	-2.397540
F	-5.198965	-0.441294	-1.853778
O	4.577015	-1.674674	-0.297277
S	5.009091	-3.077640	-0.449061
N	5.034657	-3.931963	0.926591
S	5.501219	-3.309342	2.358753
C	3.883280	-2.782232	3.154695
F	3.426305	-1.651409	2.575329
O	4.350108	-3.895287	-1.471887
C	6.793970	-2.973130	-1.036557
F	7.621240	-2.630072	-0.039494
F	7.177732	-4.147201	-1.531864
F	6.871751	-2.038476	-1.993139
O	5.973130	-4.400716	3.204356
O	6.259678	-2.046158	2.298735
F	4.068642	-2.547076	4.450851
F	2.941846	-3.735178	3.017340
H	2.688577	-7.217579	-0.377769
H	-3.198747	-2.825859	-0.190242
H	7.940239	1.624149	-1.405895
H	-0.933588	-0.974517	2.805640
H	-4.308804	-1.137459	4.805047
H	-4.173189	-0.053089	3.403267
H	-2.253471	0.147269	4.979766
H	-1.816782	-1.547480	4.690841
H	-1.585160	1.070047	1.630323
H	-0.504819	2.959469	2.797800
H	-2.224831	3.069454	2.352302
H	0.453080	0.943019	1.110886
H	2.039193	1.293388	-0.371788
H	2.762025	-0.257185	0.095088
H	3.557199	0.810677	2.250753
H	3.059843	2.390693	1.609741
H	0.784052	-1.137016	1.877738
H	-0.615521	-2.997546	0.819726
H	0.437455	-3.949354	1.895444
H	2.436491	-2.749150	0.846810
H	1.327457	-2.003075	-0.305313
H	-2.885407	2.752420	0.141032
H	-4.248249	5.655600	-2.726603
H	-2.374377	7.159188	-1.975304
H	5.437639	-0.208401	1.572019
H	7.552208	-0.192069	0.304478
H	6.137418	3.356317	-1.741158
H	2.486192	-5.135294	0.996956
H	1.902528	-7.164013	-2.764408
H	0.833624	-3.031926	-2.174766
H	-5.121859	-5.191393	2.882172
H	-4.267885	-4.994166	0.514173

H	-3.049569	-0.983781	1.430511
H	0.945015	-5.004470	-3.667808
H	-4.830349	-3.245571	4.419649
H	4.059969	3.211169	-0.359774
H	-4.491479	3.398737	-1.611775
H	-0.823340	6.343212	-0.191075
H	-0.651597	5.036181	1.699818
H	0.092982	3.936413	0.548192

[HEMim][NTf$_2$]

```
B3LYP-D3/6-31+G* calculated frequencies and intensities

monomer
======

Frequencies --   3305.6851               3338.2941               3593.0751
IR Inten    --      4.2068                  5.6121                495.7479

dimer
=====
```

c-a

```
Frequencies --   3336.1831               3635.6513               3767.5256
IR Inten    --      4.0709                288.1275                 94.9245
```

c-c

```
Frequencies --   3323.2900               3555.1016               3631.1674
IR Inten    --     18.8052                477.4433                249.9534

trimer
======
```

c-a

```
 Frequencies --   3646.1083               3661.5881               3665.5371
 IR Inten    --    395.3885                204.8542                217.9838
```

c-c

```
 Frequencies --   3436.2915               3525.7435               3580.0134
 IR Inten    --    614.1633                684.3541                471.9585

tetramer
========
```

c-a

```
 Frequencies --   3324.8246               3330.2434               3601.2754
 Frequencies --   3607.2423               3651.4873               3723.7553
 IR Inten    --      4.5022                  9.7917                529.5344
 IR Inten    --    321.8608                238.5172                 91.7938
```

c-c

```
 Frequencies --   3308.8960               3310.1447               3310.9594
 Frequencies --   3323.6965               3325.1860               3328.0909
 Frequencies --   3337.8482               3343.9655               3368.3570
 Frequencies --   3389.3314               3422.5428               3483.4410
 IR Inten    --     12.9785                627.5321                 11.8658
 IR Inten    --     11.6676                  7.6439                 14.7197
 IR Inten    --     18.3073                 36.1868                 13.7146
 IR Inten    --   1430.0905               1455.3825                624.1350
```

SI5 DSC study of thermal behavior.

The thermal behavior of 5 ILs with -OH group was studied with Mettler Toledo DSC 822e coupled with Huber TC100MT cooler. The sample was placed in the standard non-pinned aluminum pan of 40 μl volume. All handling operations with sample were carried out in glove-box under a nitrogen atmosphere (residual concentrations of oxygen 1.0 ppm and water 0.3 ppm). Pans and samples were weighted with Sartorius MSE3.6P-000-DM microbalances with the standard uncertainty of $5 \cdot 10^{-6}$ g. The calibration of Mettler Toledo DSC 822e was checked with melting behavior of the reference Indium sample. The temperature of fusion agreed with recommended value better than 0.5 K and the fusion enthalpy within 0.2 $kJ \cdot mol^{-1}$.

The temperature profile for all compounds (except [HETMA][NTf_2]) was the same - cooling down to 193 K at 10, 5 or 2 $K \cdot min^{-1}$ cooling rate and keeping for annealing for 30 minutes at this temperature. After that, the sample was heated to 373 K with a rate of 2 $K \cdot min^{-1}$, 5 $K \cdot min^{-1}$ or 10 $K \cdot min^{-1}$. For all compounds, two samples of different masses were utilized. The glass transition temperature (T_g, onset of the heat capacity change), crystallization temperature (T_c), and melting temperature (T_{fus}) were determined from DSC thermograms during the heating scans.

The sample of [HETMA][NTf_2] was solid at room temperature. Therefore, it was heated up to 373 K in order to melt the sample and derive the equilibrium crystal with good thermal contact with pan's walls during cooling down to 193 K. The other steps were left the same.

The summary of phase transitions is given in Table S1.

[HETMA][NTf$_2$] (I)

Apparently, for [HETMA][NTf$_2$] (**I**) polymorphic phases are formed during cooling. At the same time the phases are more stable than in the case of [HEMPyrro][NTf$_2$]. The low cooling rate stems in increasing the enthalpy of irreversible phase transitions and didn't shift significantly the other phase transitions.

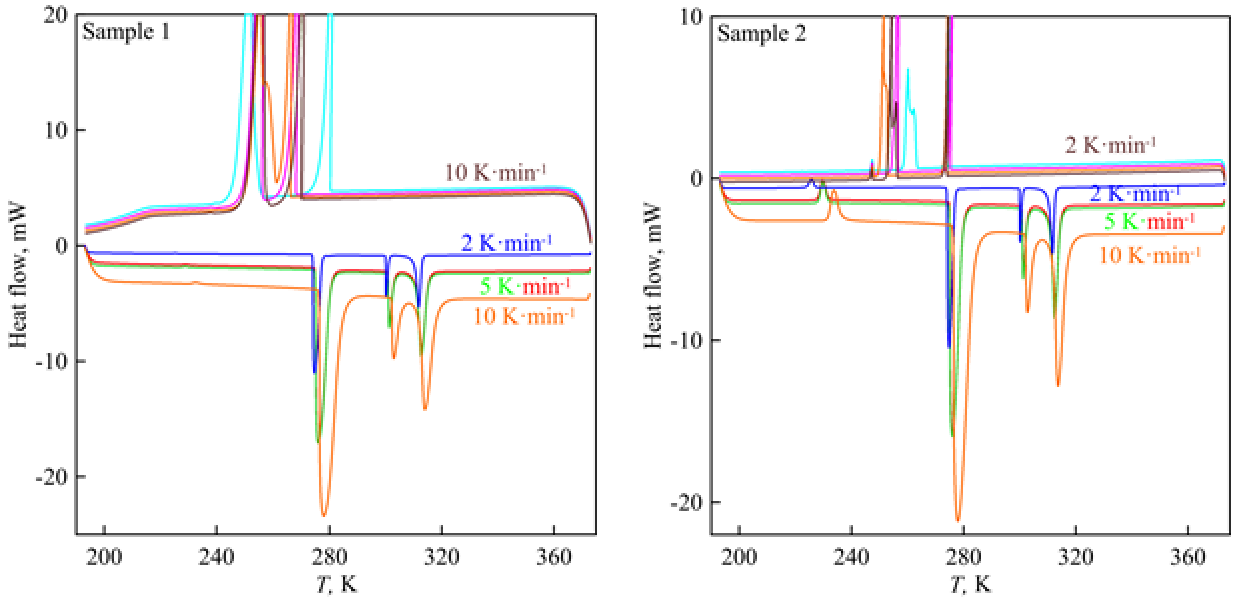

SI Fig. 2 The DSC profile for [HETMA][NTf$_2$] (**I**) samples; blue line is the heating with 2 $K \cdot min^{-1}$, green and red lines – heating with 5 $K \cdot min^{-1}$, orange line - heating with 2 $K \cdot min^{-1}$. The curves with the same heating or cooling rate are shifted for 0.1 mW for better illustration.

[HEMPip][NTf$_2$] (II)

For this IL the thermal behavior is also rather complicated. Nevertheless, no irreversible phase transitions were observed.

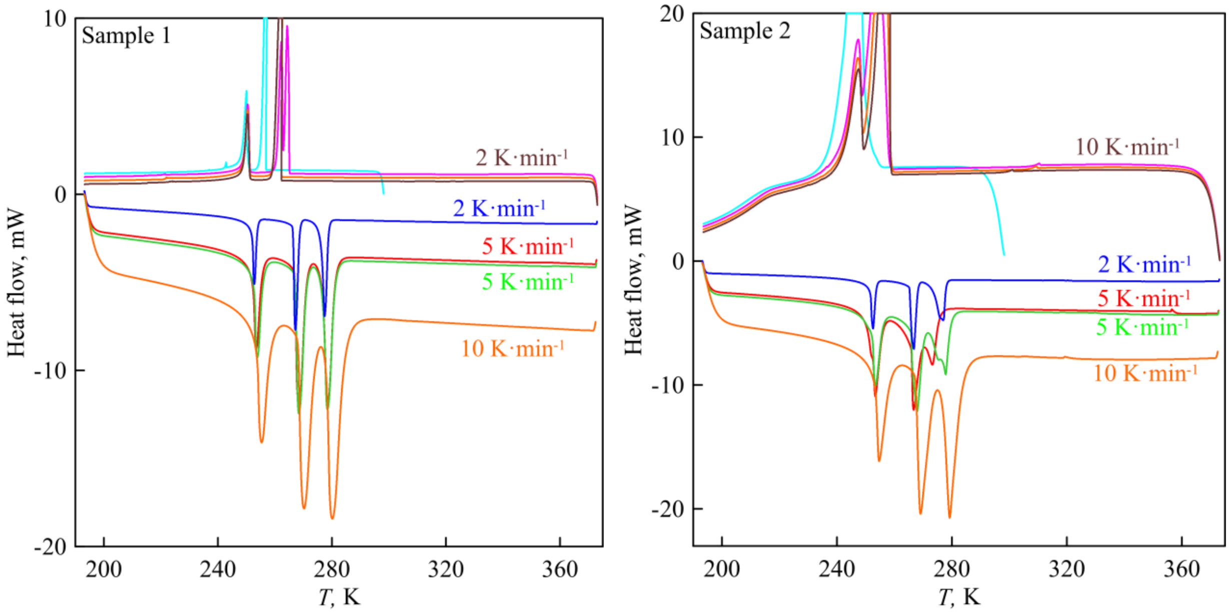

SI Fig. 3 The DSC profile for [HEMPip][NTf$_2$] (**II**) samples; blue line is heating with 2 K·min^{-1}, green and read lines – heating with 5 K·min^{-1}, orange line - heating with 10 K·min^{-1}. The curves with the same heating or cooling rate are shifted for 0.1 mW for better illustration.

It might be admitted that the temperature of fusion and of the one of solid to solid phase transition are shifted in two samples. This can be due to the complications in selecting the baseline, while peaks are overlapped.

$[HEMPyrro][NTf_2]$ (III)

Apparently, $[HEMPyrro][NTf_2]$ (**III**) has few polymorphic phases below 273 K. The number of phase transitions and their temperatures are significantly dependent on the heating rate. The heating with 2 K·min^{-1} better corresponds to the conditions of IR analysis of the sample (see Fig. 2c).

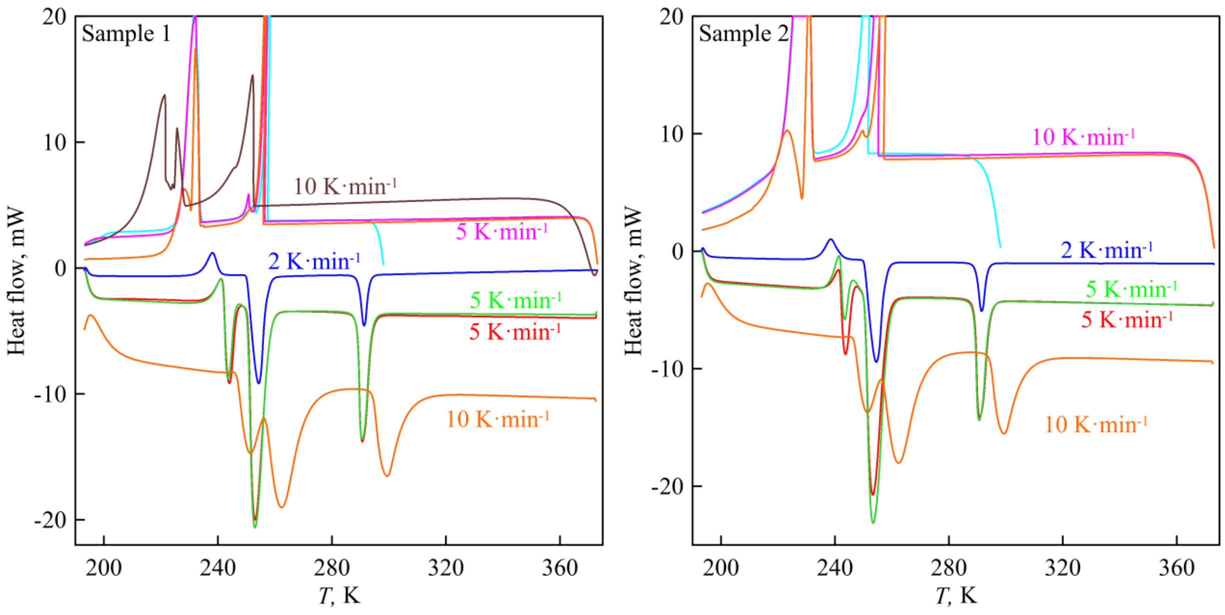

SI Fig. 4 The DSC profile for $[HEMPyrro][NTf_2]$ (**III**) samples; blue line is heating with 2 K·min^{-1}, green and red lines – heating with 5 K·min^{-1}, orange line – 10 K·min^{-1}. The curves with the same heating or cooling rate are shifted for 0.1 mW for better illustration.

[HEPy][NTf2] (IV)

No phase transition was observed during cooling down to 193 K. During heating the glass transition at 200.4 K was observed and reproduced for both samples. No fusion or crystallization peak has been seen for both samples at heating rates from 2 to 10 K·min^{-1}.

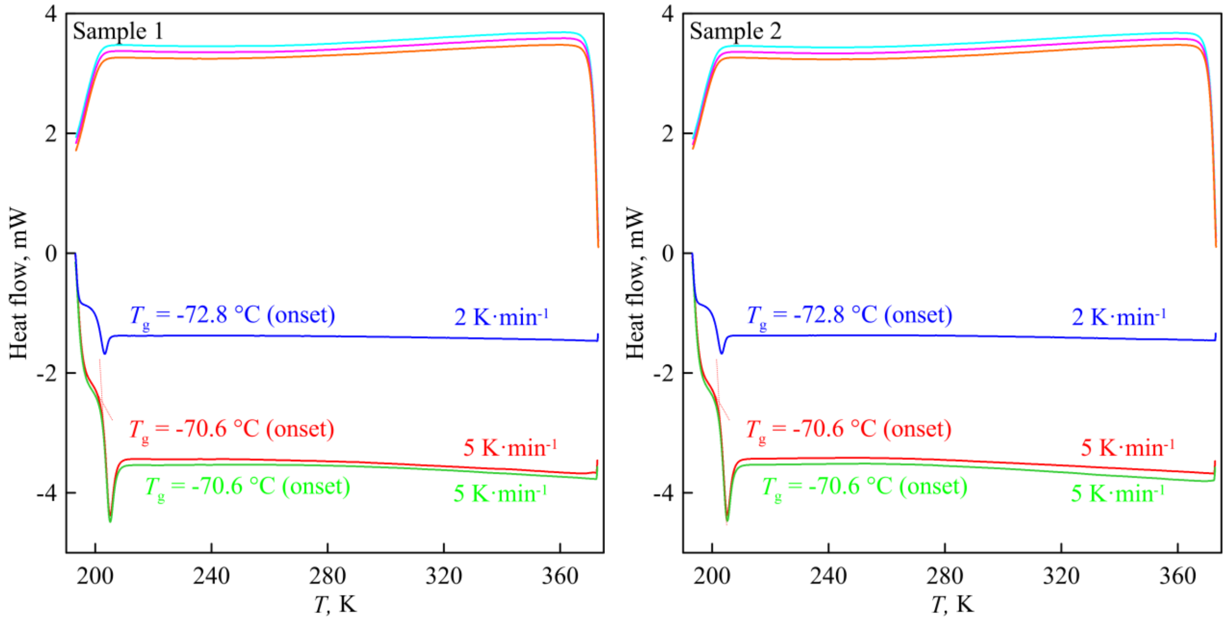

SI Fig. 5 The DSC profile for [HEPy][NTf2] (**IV**) samples; blue line is the heating with 2 K·min^{-1}, green and red lines – heating with 5 K·min^{-1}. No peak of fusion was observed. The curves with the same heating or cooling rate are shifted for 0.1 mW for better illustration.

[HEMIm][NTf$_2$] (V)

The thermal behavior of [HEMIm][NTf$_2$] is very close to that of [HEPy][NTf$_2$]. The sample was prepared by cooling with 2 and 10 K·min^{-1} rate. In both cases, no crystallization peak was observed. On heating from 193 to 373 K only glass transition in the interval 195 – 198 K is recorded. Due to the device limitations, the more precise determination of glass transition temperature is not possible. No melting peak is seen independently on the rate of sample cooling.

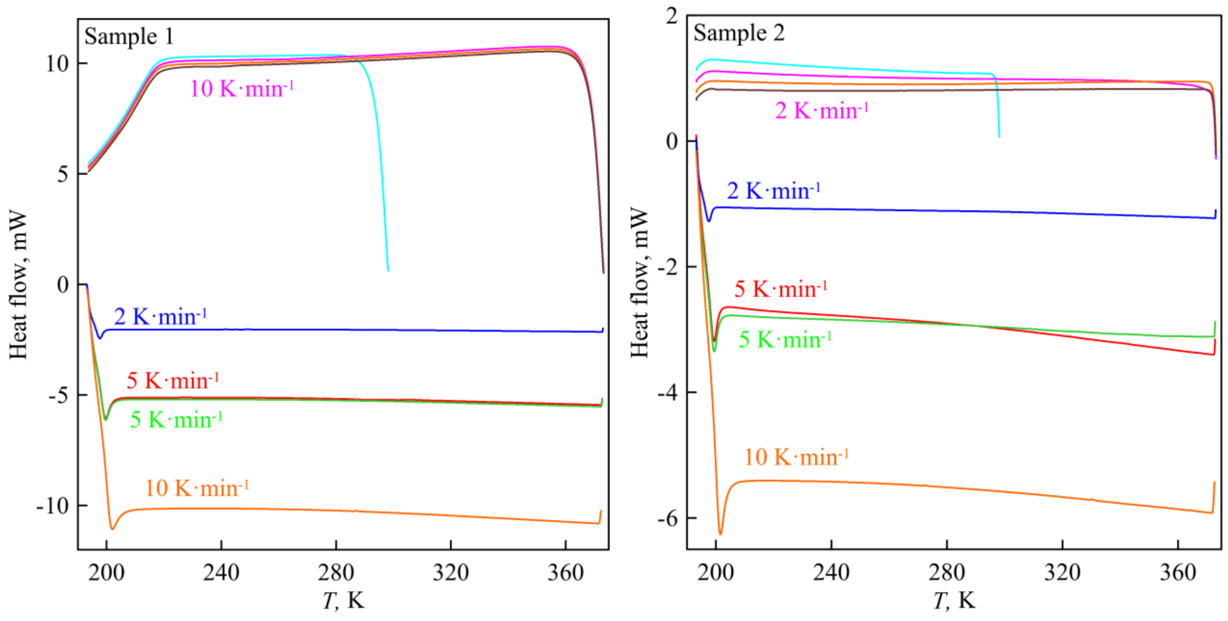

SI Fig. 6 The DSC profiles for [HEMIm][NTf$_2$] (**V**) samples; orange line corresponds to heating with 10 K·min^{-1}, green and red lines – heating with 5 K·min^{-1}, blue line - heating with 2 K·min^{-1}. Sample 1 was cooled with 10 K·min^{-1}, sample 2 with 2 K·min^{-1}. The curves with the same heating or cooling rate are shifted for 0.1 mW for better illustration.

Table S3 The thermodynamic parameters of observed phase transitions for studied ILs **I - IV**.

	Ionic liquid	Phase transition	T_{trs}, K	$\Delta_{trs}H^{o}_{m}$, kJ·mol^{-1}	Comments
**I**	[HETMA][NTf_2]	irreversible solid - solid	227.8 ± 0.9	-0.035 ± 0.005<br>-0.70 ± 0.09	cooling at 10 K·min^{-1}<br>cooling at 2 K·min^{-1}
		reversible solid-solid	274.6 ± 0.9	11.6 ± 0.2	
		reversible solid-solid	300.5 ± 0.8	1.4 ± 0.2	
		solid - liquid	310.4 ± 0.3	4.53 ± 0.1	
**II**	[HEMPip][NTf_2]	solid - solid	251.9 ± 1.3	2.7 ± 0.3	
		solid - solid	266.7 ± 1.5	4.2 ± 0.2	
		solid – liquid	276.6 ± 0.9	4.4 ± 0.3	
**III**	[HEMPyrro][NTf_2]	irreversible solid - solid	235 ± 0.1	-2.9 ± 0.1	
		reversible solid – solid 1	250.2 ± 0.3	13.5 ± 0.1	Solid 1 and solid 2 are two coexisting phases with different temperature and enthalpy of fusion.
		reversible solid – solid 2	251.6 ± 0.9	11.6 ± 0.7	
		solid 1 - liquid	289.7 ± 1.0	3.9 ± 0.1	
		solid 2 - liquid	288.7 ± 0.4	5.1 ± 0.1	
**IV**	[HEPy][NTf_2]	glass - liquid	200.4 ± 0.1	-	Small relaxation peak is observed at T_g
**V**	[HEMim][NTf_2]	glass - liquid	195 ± 2	-	

SI6 Single crystal preparation and X-ray Determination:

The crystal growth of [HEMPip]NTf_2 (**II**) was performed in a low-temperature mounting device under an inert N_2 atmosphere, cooled with an ENRAF NONIUS FR558-S cooling-unit: Small droplets of the pure substance were placed in dry Fomblin Y perfluoroether (Sigma-Aldrich) at ambient temperature. X-ray quality crystals were obtained by slow cooling (approx. 5 °C min^{-1}) to 173 K. A three-fold (non-merohedrally) twinned specimen was selected and mounted at this temperature. The sample was cooled to 123(2) K during measurement. The data was collected on a Bruker D8 QUEST diffractometer using Mo Kα radiation (λ = 0.71073).

The de-twinned data of one twin domain (*TWINABS*)[Fehler! Textmarke nicht definiert.] was used for structure refinement. The structure was solved by direct methods (*SHELXS-2013*)[1] and refined by full-matrix least squares procedures (*SHELXL-2013*).[Fehler! Textmarke nicht definiert.] A semi-empirical absorption correction was applied (*SADABS*).[2] All non-hydrogen atoms were refined anisotropically, hydrogen atoms were included in the refinement at calculated positions using a riding model. Torsion angles of all methyl groups, as well as hydroxyl groups were allowed to refine. Further details can be found in the cif-file (CCDC 1540883).

[1] a) G. M. Sheldrick, *Acta. Cryst.* **2008**, *A64*, 112 – 122; b) G. M. Sheldrick, *Acta Cryst.* **2015**, *C71*, 3 – 8.

[2] L. Krause, R. Herbst-Irmer, G. M. Sheldrick, D. Stalke, *J. Appl. Crystallogr.* **2015**, *48*, 3 – 10.

Table S2. Crystallographic details of [HEMPip]NTf_2.(**II**)

Chem. Formula	$C_{10}H_{18}F_6N_2O_5S_2$
Form. Wght. [g mol^{-1}]	424.38
Color	Colorless
Cryst. system	Monoclinic
Space group	*C c*
a [Å]	14.8992(9)
b [Å]	14.8514(9)
c [Å]	8.6275(5)
α [°]	90
β [°]	117.184(2)
γ [°]	90
V [Å^3]	1698.2(2)
Z	4
$\rho_{calc.}$ [g cm^{-3}]	1.660
μ [mm^{-1}]	0.401
$\lambda_{MoK\alpha}$ [Å]	0.71073
T [K]	123(2)
Measured reflections	2185
Independent reflections	2185
Reflections with $I > 2\sigma(I)$	1496
$R_{int.}$	0.0269
F(000)	872
R_1 (R [$F^2 > 2\sigma(F^2)$])	0.0382
wR_2 (F^2)	0.0698
GooF	0.931
Parameters	378
CCDC #	1540883
